# COVID-19 pandemic in Africa: Is it time for water, sanitation and hygiene to climb up the ladder of global priorities?

**DOI:** 10.1016/j.scitotenv.2021.148252

**Published:** 2021-10-15

**Authors:** P. Marcos-Garcia, C. Carmona-Moreno, J. López-Puga, A.M. Ruiz-Ruano García

**Affiliations:** a European Commission–Joint Research Centre, via E. Fermi 2749, 21027 Ispra, VA, Italy; b Universidad de Granada, Campus de Cartuja, s/n, 18071 Granada, Spain

**Keywords:** WASH, Respiratory infections, COVID-19, Migrant remittances, Official development assistance, Africa

## Abstract

In the current pandemic context, it is necessary to remember the lessons learned from previous outbreaks in Africa, where the incidence of other diseases could rise if most resources are directed to tackle the emergency. Improving the access to water, sanitation and hygiene (WASH) could be a win-win strategy, because the lack of these services not only hampers the implementation of preventive measures against SARS-CoV-2 (e.g. proper handwashing), but it is also connected to high mortality diseases (for example, diarrhoea and lower respiratory infections (LRI)). This study aims to build on the evidence-based link between other LRI and WASH as a proxy for exploring the potential vulnerability of African countries to COVID-19, as well as the role of other socioeconomic variables such as financial sources or demographic factors. The selected methodology combines several machine learning techniques to single out the most representative variables for the analysis, classify the countries according to their capacity to tackle public health emergencies and identify behavioural patterns for each group. Besides, conditional dependences between variables are inferred through a Bayesian network. Results show a strong relationship between low access to WASH services and high LRI mortality rates, and that migrant remittances could significantly improve the access to healthcare and WASH services. However, the role of Official Development Assistance (ODA) in enhancing WASH facilities in the most vulnerable countries cannot be disregarded, but it is unevenly distributed: for each 50–100 US$ of ODA per capita, the probability of directing more than 3 US$ to WASH ranges between 48% (Western Africa) and 8% (Central Africa).

## Highlights

• Most African countries are still highly vulnerable to infectious respiratory diseases.
• Low access to WASH services increases the probability of having a high LRI mortality.
• Remittances improve the access to WASH and healthcare services at household level.
• Vulnerable countries depend on ODA for WASH access, but it is unevenly distributed.
• Well-targeted ODA could alleviate the effects of reduced remittances due to COVID-19.

## Graphical abstract

**Figure:**
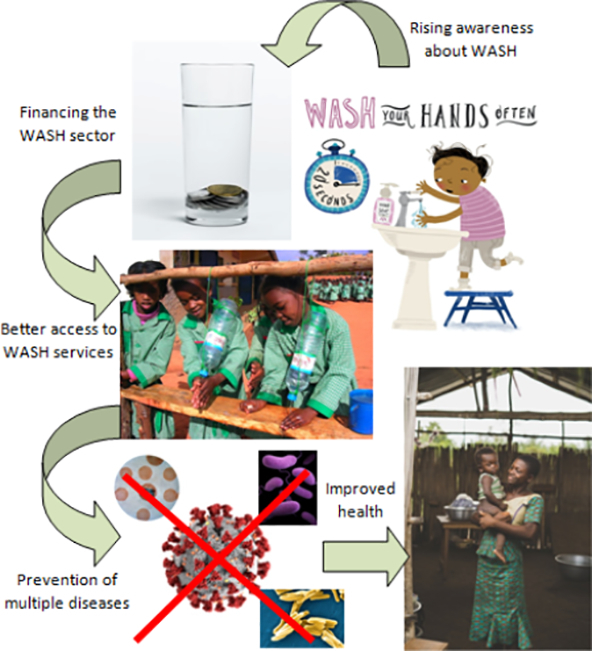

## 1 Introduction

Handwashing with soap and water for at least 20 s is a well-known, effective preventive measure to curb the spread of infectious diseases, and it has been highly encouraged in the context of the COVID-19 pandemic (WHO/UNICEF, 2020). However, its worldwide implementation is hampered by the lack of access to water, sanitation and hygiene (WASH) services in regions such as sub-Saharan Africa, where one third of the population do not have access to an improved water source and the prevalence of handwashing with soap at critical times (after defecation and before eating) has been estimated to be just 14% (Roche et al., 2017). Lack of access to WASH services has been widely recognized as a risk factor involved in the development of multiple diseases, including diarrhoea, schistosomiasis, protein-energy malnutrition or lower respiratory infections (LRI) (Prüss-Ustün et al., 2014, Prüss-Ustün et al., 2019; Troeger et al., 2018; Stanaway et al., 2018). Concretely, LRI (which include pneumonia, as well as acute bronchitis, bronchiolitis, influenza and whooping cough) were the deadliest communicable diseases in 2016, causing 3 million deaths worldwide, and the leading cause of death in low-income countries (WHO, 2020c). In this regard, several authors have pointed out that a significant percentage of LRI could be avoided by proper handwashing (Rabie and Curtis, 2006; Azupogo et al., 2019). The evidence supporting this link between other respiratory infections and WASH services could be a valuable source of information in the case of COVID-19 pandemic.

Integrating the improvement of WASH services within the international response to the pandemic could be a win-win strategy, because it would also be able to reduce the mortality and morbidity of other prevalent diseases (e.g. malaria, diarrhoea, bacterial infections or tuberculosis), which are treatable and partly preventable. Besides, if a better access to WASH services is accompanied by the implementation of hygiene promotion multimodal strategies (Irehovbude and Okoye, 2020), the pandemic could still trigger positive and long standing behavioural changes to fight other infectious diseases in the continent (Wadoum and Clarke, 2020). However, lessons learned from previous outbreaks show that the incidence of other diseases could rise if most resources are directed to tackle the emergency. For example, Walker et al. (2015) estimated that the 2014 Ebola outbreak led to 3.5 million of additional malaria untreated cases in Guinea, Sierra Leone and Liberia, and multiple authors have already warned about the potential of the COVID-19 pandemic to reverse two decades of progress against malaria (Weiss et al., 2020; Nghochuzie et al., 2020; Sherrard-Smith et al., 2020) or other prevalent neglected tropical diseases (NTDs) in Africa (Molyneux et al., 2020). The importance of WASH as part of the pandemic response has been already recognized by several world leaders through a call for its prioritisation (SWA, 2020). In addition, the EU Global Response to COVID-19 (EC, 2020) explicitly states the necessity to provide immediate targeted support in the sectors of health, WASH and logistics. However, although there is a broad consensus about the health and social benefits of WASH interventions in humanitarian crises, D'Mello-Guyett et al. (2018) pointed out the paucity of rigorous evidence to guide a more effective and efficient investment in WASH as part of an emergency response. In this regard, Howard et al. (2020) performed a comprehensive review on WASH and COVID-19 and suggested that WASH should be integrated both into preparation plans and with other sectors, along with more financing and a better use of financing instruments.

Long-term planning is essential for the development of sustainable WASH services. Both Millennium Development Goals (MDGs) and Sustainable Development Goals (SDGs) set specific targets for WASH in the international agenda, and substantial achievements have been made since 1990. However, progress has been uneven across different regions, between urban and rural areas, and between rich and poor. According to Fuente et al. (2020), in many sub-Saharan African countries, expected economic growth alone will not be sufficient to eliminate WASH-related mortality or the economic losses associated with poor access to water and sanitation infrastructure by 2050. Moreover, it is admitted that the current level of WASH financing would not be enough to close the gap between SDG6 aspirations (universal access to safe and affordable drinking-water, adequate sanitation and hygiene) and national realities. Sources of funds for WASH include: 1) taxes from individuals and businesses; 2) transfers such as overseas aid, remittances or market interest rate lending; and, 3) tariffs paid by households, businesses and governments. According to WHO and UN-Water (2017), some of the main issues in WASH financing are: lack of mechanisms to cover operational financial gaps (household tariffs are often insufficient), leading to deferred maintenance, deterioration of assets and increased failure rate; uncertainty of future Official Development Aid (ODA) investments (WASH ODA commitments have declined since 2012) and; lag between policy priority and implementation regarding the provision of WASH services to vulnerable groups.

For these reasons, the current investments to fight against the COVID-19 pandemic should be considered as an opportunity for the WASH sector to climb up the ladder of priorities at global level, integrating the crises response of the international community within the long-term framework set by SDG6. This paper provides scientific insights on two main questions regarding African countries: what are the relationships between respiratory infections, WASH and other socioeconomic variables at national level? and; which countries could be more vulnerable to the current pandemic, in order to prioritize WASH interventions?

## 2 Materials and methods

### 2.1 Materials

For this study, annual data of several variables at national level were retrieved from different sources (Table 1 in Supplementary material), using 2000–2016 as reference period when data availability allowed it.

**Table 1:** Factor loadings of the three first components of the PCA (69% of the total variance explained).

Variables	F1	F2	F3
Pop_dens	0.28	−0.31	**−0.66**
Pop_urb	**0.64**	0.10	0.42
Urb_aglom_1M	0.05	−0.17	**0.74**
Life_expect	**0.81**	**−0.47**	0.09
Health_exp_cap	**0.71**	**0.57**	−0.09
HAQ_index	**0.93**	0.01	0.02
Mort_rate	**−0.73**	**0.42**	−0.11
Basic_sanit	**0.86**	0.10	−0.03
Basic_drink	**0.86**	0.07	−0.02
Basic_hyg	**0.80**	−0.01	0.27
GDP_cap	**0.61**	**0.51**	0.03
Remit_cap	**0.61**	−0.36	−0.30
ODA_cap	0.20	−0.29	**−0.41**
Pop_age	**0.89**	0.10	−0.06
Pop_HIV	0.01	**0.81**	−0.25
Mort_LRI	**−0.81**	0.00	−0.05
LRI_un5	**−0.86**	−0.06	0.17

Factor loadings with the highest values for each component (absolute value of more than 0.4).

#### 2.1.1 Population dynamics

Data on total population and median age of the total population for each country were obtained from UN (2019), while data on population density, urban population and population living in big cities (more than 1 million of inhabitants) were retrieved from the World Bank DataBank (https://databank.worldbank.org/).

#### 2.1.2 Healthcare related variables

Data on life expectancy at birth and health expenditure were obtained from the World Bank Databank. We also used the Healthcare Access and Quality Index (HAQ) estimated by Fullman et al. (2018), available through the Institute for Health Metrics and Evaluation (IHME) Global Health Data Exchange, http://ghdx.healthdata.org/).

#### 2.1.3 Economic variables and WASH sources of funds

Data on Gross Domestic Product (GDP), migrant remittance inflows and net official development assistance and official aid received (ODA) were retrieved from the World Bank DataBank. The economic data of the Federal Reserve Bank of St. Louis (https://fred.stlouisfed.org/) were used as an additional source of information on these variables (to complete data gaps in the primary source). Regarding WASH financing, data on gross ODA aid disbursement for water supply and sanitation were obtained from the World Bank for the available period (2003−2011). Finally, data on the expenditure of migrant remittances at the household level for four African countries (Burkina Faso, Kenya, Nigeria and Uganda) were retrieved from the World Bank Migration Household Surveys (University of Ouagadougou, 2009; University of Nairobi, 2009; Zibah Consults Limited, 2009; Makerere Statistical Consult Limited, 2010).

#### 2.1.4 Disease burden and mortality estimates

Data on highly prevalent diseases in African countries (malaria and tuberculosis) and on diseases in which the lack of access to WASH services has been recognized as a risk factor (diarrhoea, LRI, protein-energy malnutrition and schistosomiasis) were extracted from the latest WHO country-level cause-specific mortality estimates (WHO, 2018). Data on estimated number of people living with HIV in each country were retrieved from the WHO Global Health Observatory data repository (https://apps.who.int/gho/data).

#### 2.1.5 Potential risk factors involved in LRI

Medical literature identifies lack of WASH services and air pollution as risk factors in the development of LRI, concretely: lack of handwashing facilities with soap and water and clean cooking fuels, second hand smoking and exposure to PM 2.5 air pollution (e.g. Troeger et al., 2018). Data on population using at least basic drinking water services, population using at least basic sanitation services and population with access to basic handwashing with soap and water were obtained from the WHO/UNICEF Joint Monitoring Programme for Water Supply, Sanitation and Hygiene (JMP). In order to complete the data on basic handwashing, we made estimations using access to basic drinking and basic sanitation services as explanatory variables in a multivariate regression model. Data on population access to clean cooking fuels and technologies and three variables related to PM2.5 pollution (percentage of population exposed to levels exceeding WHO Interim Target-1/ Target 2 values and mean annual exposure (μg/m^3^) were obtained from the World Bank DataBank. Regarding smoking prevalence, the World Bank DataBank was used as a primary source and the data collected by Ng et al. (2014) as a secondary source (which could be accessed through the IHME Global Health Data Exchange).

#### 2.1.6 COVID-19 data

Data on daily COVID-19 tests (Hasell et al., 2020), deaths and confirmed cases at national level were retrieved from Our World in Data (https://ourworldindata.org/coronavirus-testing).

### 2.2 Methods

**Fig. 1:**
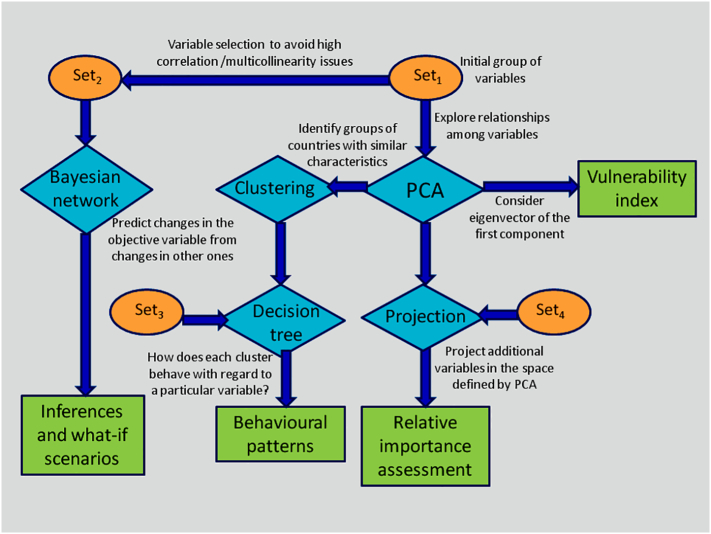
Overall approach scheme.

The selected approach (Fig. 1) involved several steps: in first place, a Principal Component Analysis (PCA) was performed on an initial group of variables (Set_1_) to explore the relationships between variables. PCA components were used with different purposes: 1) the first component was used to obtain a potential vulnerability index for each country and year; 2) the relative importance of several risk factors and prevalent diseases (Set_4_) was assessed by projecting them onto the space defined by the two first components; 3) the first four components of the PCA were used to find out clusters of countries with similar characteristics, through the Hierarchical Clustering on Principal Components (HCPC) algorithm. In the next step, the behaviour of each cluster of countries in relation to other variables (Set_3_) was explored by means of a classification decision tree algorithm.

Removing highly correlated variables (which often represent a same underlying concept) could avoid problems when it comes to model fitting and the interpretation of results (e.g. a Bayesian network that includes highly correlated variables could become densely connected and inferences could become computationally expensive). For this reason, we analyzed the correlation matrix and the Variance Inflation Factor (VIF) (Marquardt, 1970) to detect potential multicollinearity issues and select a representative subset of variables from Set_1_ (Set_2_) as nodes for a Bayesian network (BN). The BN allowed us both to obtain conditional probabilities and to explore what-if scenarios (assessing potential impacts induced by changes in one or more variables and their subsequent propagation through the BN). The four groups of variables were summarized in Table 2 in Supplementary material. Finally, a brief analysis of the COVID-19 testing capacity of African countries, daily rate of confirmed cases and deaths is included to provide insights into the potential limitations of the selected methodology and the current knowledge of the evolution of the pandemic in the continent.

#### 2.2.1 Principal component analysis and potential vulnerability index

First, we selected an initial group of variables (Set_1_) that could have a potential relevant role regarding national vulnerability to COVID-19: population density, urban population, population living in big cities (more than 1 million inhabitants), median age of the total population, life expectancy at birth, health expenditure, HAQ index, GDP per capita, migrant remittance inflows per capita, net ODA per capita, LRI total mortality rate, LRI mortality rate in children under 5 years, HIV infected rate and access to basic WASH services (drinking water, sanitation and handwashing with soap and water). Next, we removed the effects of population size from the original variables by transforming some of the variables to per capita ones (e.g. Gross Domestic Product (“GDP_cap”), migrant remittance inflows (“Remit_cap”) or ODA (“ODA_cap”)) and mortality rates (e.g. “LRI_deaths” to “Mort_LRI”). The rows with missing values were removed from the dataset and then the selected variables were standardized to have mean = zero and standard deviation = 1.

The initial set of selected variables (V_1_) described multiple characteristics for each country. However, some of these properties could be correlated and redundant. PCA is able to reduce the dimensionality of a particular system without reducing the number of features of the data. Instead of selecting some characteristics and discarding others, it constructs some new variables (as linear combinations of the initial ones) which turn out to summarize the whole array of system properties in an appropriate way. Hence, a PCA was performed on Set_1_ (concretely, a singular value decomposition of the centered and scaled data matrix) and the first components were analyzed.

To obtain a potential vulnerability index for each country and year (Eq. (1)), we used the eigenvector corresponding to the first component as proposed by Carmona-Moreno and Marcos-Garcia (2020). The index was re-scaled from 0 (low vulnerability) to 1 (high vulnerability). We also projected the variables related to risk factors and prevalent diseases (Set_4_) in the space defined by the two first components, in order to assess their relative importance for the analysis.

(1) $$\;\mathrm{Vulnerability index}=\sum_{i=1}^{n}{\mathrm{sign}}_{i}*v_{i}^{2}*X_{i}$$

where:

– X_i_: variable i.
– v_i_: eigenvector element of first PCA component corresponding to X_i_.
– Sign_i_: sign of the eigenvector element v_i_.

#### 2.2.2 Cluster analysis

To identify groups of countries with similar profiles, we used the Hierarchical Clustering on Principal Components (HCPC) implemented in the “FactoMineR” R package (Lê et al., 2008), which allows to perform a hierarchical classification on the principal components of a factor analysis and then a partitioning clustering (particularly the K-means method). We applied this algorithm to the four first components of the PCA.

#### 2.2.3 Relationship between LRI mortality, access to WASH services and air pollution

A classification decision tree was used to predict the cluster to which a country belongs (dependent variable) using a set of variables as explanatory ones and the “rpart” R package (Therneau et al., 2019). Clusters were obtained in Section 3.3 for the year 2016. In first place, we estimated the correlation coefficients between the risk factors involved in LRI (access to WASH services and clean cooking fuels, prevalence of smokers and exposure to PM 2.5) and the LRI mortality rates in adults (“LRI_ov5”) and children (“LRI_un5”) (Set_3_). Secondly, the correlation coefficients were coded using dummy variables which took the value 0 if the correlation was over 0.7 and the sign was negative (in the case of access to WASH services and clean cooking fuels) or positive (in the case of smokers prevalence and variables related to PM2.5). For each country and age group, the values of the dummy variables were added. Next, the classification decision tree algorithm is used to predict the country cluster depending on the aggregated values of the dummy variables for both population segments (LRI_ov5 and LRI_un5).

#### 2.2.4 Bayesian network

Bayesian networks (BNs) are probabilistic graphical models able to represent a set of variables and their conditional dependences by means of a Directed Acyclic Graph (DAG). Here, a BN of ten nodes (corresponding to the variables included in Set_2_ plus the ODA per capita specifically directed to the WASH sector) was built using the “bnlearn” R package (Scutari, 2010). Structure learning was performed using the continuous variables and several algorithms: constraint-based (Grow-Shrink and Incremental Association Markov Blanket), score-based (Hill Climbing and Tabu Search) and hybrid (Max-Min Hill Climbing). Unlikely arcs according to prior expert knowledge were blacklisted in order to avoid their appearance in the final structure. This structure was assessed through several performance indicators and parameters were fitted using Maximum Likelihood estimates. Next, continuous variables were discretized in 3 categories (considering equal frequency intervals) to analyze their conditional probabilities according to the selected structure. Finally, we used the “BayesNetBP” R package (Yu et al., 2020) to explore what-if scenarios by adding hypothetical new information to the BN, propagate it (belief propagation) and make queries about how the probabilities change according to the new information (probabilistic reasoning).

## 3 Results

### 3.1 Dimensionality reduction

Table 1 shows the factor loadings of the three first components of the PCA (F1), which explained 48%, 12% and 9% of the total variance respectively. It could be observed that the first component is mainly related to development (variables positively correlated with this component are related to healthcare, WASH services, urbanization rates, life expectancy and economy, whereas the ones related to mortality are negatively correlated). Here, it is interesting to point out that the factor loading of the migrant remittance per capita is equal to the GDP per capita one, suggesting a relevant role of this variable. The second component is mainly related with the incidence of HIV (positive correlations include the percentage of population with HIV, GDP per capita, health expenditure per capita and the total mortality rate, whereas it is negatively correlated with life expectancy. Finally, the third component seems to be mainly related to population distribution (people living in big cities is positively correlated, while population density and ODA are negatively correlated).

As the first component is the most interesting one for the purpose of our analysis, we assessed the potential differences at regional level by repeating the PCA for each African region (Table 3 in Supplementary material). According to our results, ODA per capita seems to be very relevant in Western Africa (factor loading of 0.77), but its relative importance in Eastern Africa and Central Africa is lower (0.32 and 0.39, respectively), whereas it is not significant in Southern and Northern Africa. This fact is coherent with the amount of gross ODA for WASH received during the period 2003–2011: Western Africa received more than 4 US$ per capita, while the mean amount at the continental level was 3 US$ and in Central and Southern Africa was significantly lower (2.2 and 2.6 US$, respectively).

**Fig. 2:**
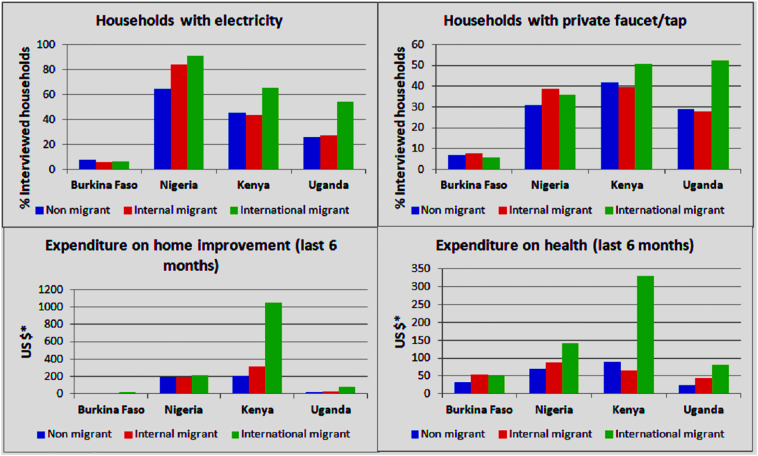
Household conditions and average expenditure depending on household type in 2009 *Considering the average official exchange rate per USD for year 2009 (when the interviews took place).

Besides, the role of the remittances appears to be more significant in Western and Eastern Africa (0.89 and 0.71, respectively) than in the other regions. In this regard, the analysis of the Migration Household Surveys in two Western African countries (Burkina Faso and Nigeria) and two Eastern African ones (Kenya and Uganda), shows that the household conditions are significantly better if there is an international migrant member in the household (e.g. higher access to electricity, existence of a separate room for cooking or a private faucet/tap). Moreover, in general these households expend more on health and home improvement than the households with non-migrant members or with internal migrant ones. However, there are differences at country level: in Burkina Faso, although the expenditure in household improvement is much higher in international migrant households (13.3 US $ on average during the last 6 months) than in non-migrant ones (2.9 US $), it seems still insufficient for effectively enhancing household conditions. Besides, although Nigeria is one of the main recipients of migrant remittances in Africa, the differences between the three household types with regard to the primary source of drinking water are not so pronounced as in other countries, such as Kenya (Fig. 2; Figs. 1 and 2 in Supplementary material).

Regarding the role of other variables in the first component at regional level, it could be observed that the percentage of people living in big cities shows a correlation lower than |0.6| in all regions. Population density is highly and positively correlated with this component in Eastern Africa (0.74), while it is negatively correlated in Southern Africa (−0.66). Besides, the percentage of population affected by HIV only shows a high factor loading in Northern Africa (−0.76). Regarding the rest of the variables, they generally show a high factor loading and a similar behaviour across regions.

### 3.2 Potential vulnerability index

**Fig. 3:**
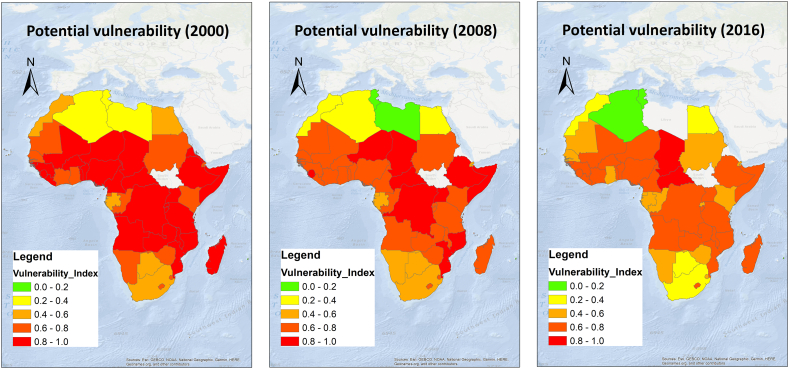
Potential vulnerability index at national level *South Sudan – No historical data for a significant analysis and classification. Lybia - no data from 2011 in advance.

Fig. 3 shows the potential vulnerability index for years 2000, 2008 and 2016. This index represents the annual state of the play at the country level for the selected socioeconomic factors and the LRI mortality rate. Therefore, highly vulnerable countries show weak healthcare systems and poor access to WASH services, low life expectancy, urbanization rates and migration remittance inflows and high mortality due to LRI. By contrast, less vulnerable countries are characterized by stronger healthcare systems and better access to WASH services, higher life expectancy, urbanization rates and migration remittance inflows and lower mortality due to LRI. It is possible to observe that throughout the reference period, the estimated vulnerability has decreased across the continent, although some countries (e.g. *Niger*, Chad or the Central African Republic) still show high values of the index.

### 3.3 Country profiles

**Fig. 4:**
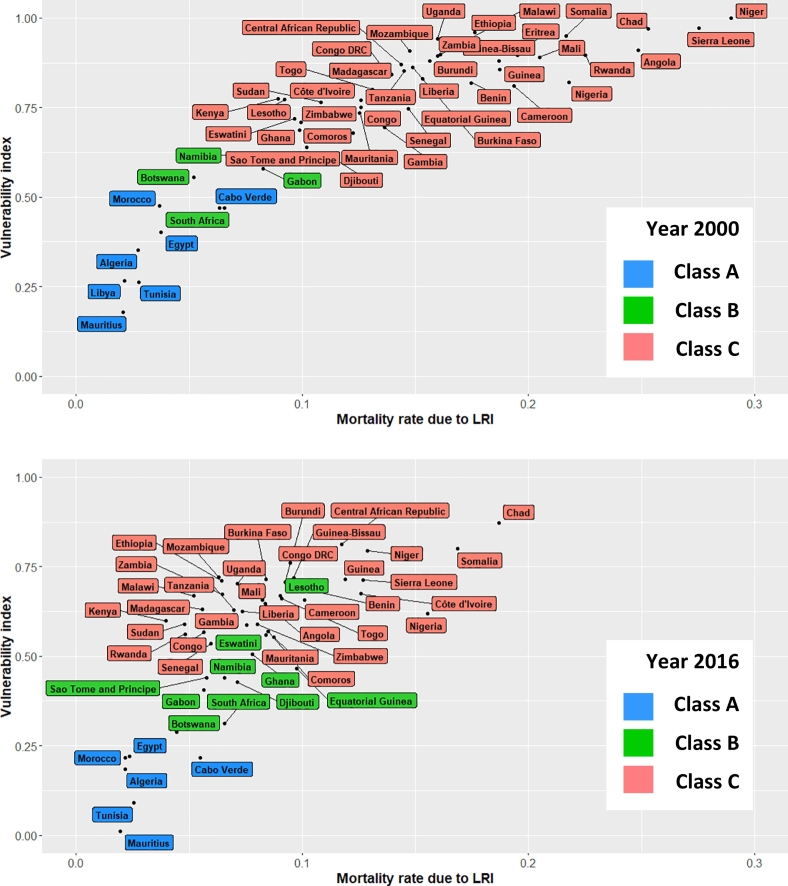
Clusters of countries for years 2000 and 2016 according to the HCDC algorithm.

The hierarchical cluster analysis suggests that African countries could be divided in three different groups according to its potential capacity to address public health emergencies, although a value of the Silhouette coefficient (Rousseeuw, 1987) of 0.21 suggests some overlapping between clusters. However, it is necessary to note that the classification of some countries changed from the beginning of the period (year 2000) to year 2016 according to the temporal evolution of the selected variables (Fig. 4):

The three groups of countries identified for the last year with complete data (2016 in most cases, except for some countries such as Libya) are the following ones:

– **Class A-2016** (low values of the potential vulnerability index, best position to tackle public health emergencies): Algeria, Cabo Verde, Egypt, Mauritius, Morocco, Seychelles, Tunisia.
– **Class B-2016** (medium values of the potential vulnerability index, intermediate capacity countries): Botswana, Djibouti, Equatorial Guinea, Eswatini, Gabon, Ghana, Lesotho, Namibia, Sao Tome and Principe, South Africa.
– **Class C-2016** (high values of the potential vulnerability index, most vulnerable countries): Angola, Benin, Burkina Faso, Burundi, Cameroon, Central African Republic, Chad, Comoros, Congo, Democratic Republic of the Congo, Côte d'Ivoire, Ethiopia, Gambia, Guinea, Guinea-Bissau, Kenya, Liberia, Madagascar, Malawi, Mali, Mauritania, Mozambique, Niger, Nigeria, Rwanda, Senegal, Sierra Leone, Somalia, Sudan, Tanzania, Togo, Uganda, Zambia, Zimbabwe.

### 3.4 Relative importance of other variables

**Fig. 5:**
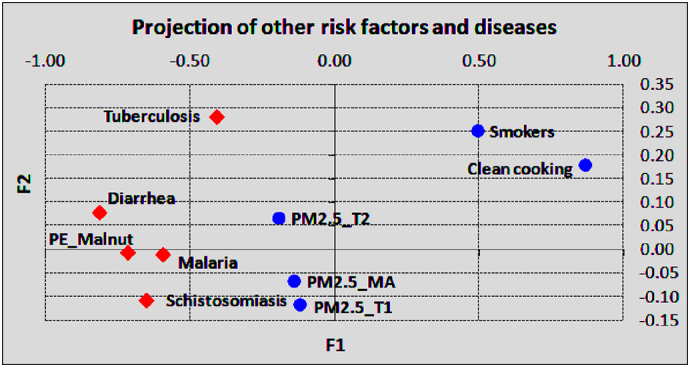
Factor loadings of the projected variables in the space defined by V_2_. F1 (48%) and F2 (12%) explain 60% of the variability associated to the variables.

Additional variables (mortality due to malaria, tuberculosis, diarrhoea, schistosomiasis and protein-energy malnutrition, access to clean cooking fuels, smoker prevalence and PM2.5) were projected in the feature space defined by the two first components using a projection matrix constructed from the coordinates of the variables obtained through the PCA. According to Fig. 5, the most significant projected variables on the axis defined by the first component are the mortality due to diarrhoea (which is strongly correlated to the access to WASH services) and the access to clean cooking fuels (which is an important risk factor in the development of LRI). On the other hand, air pollution due to PM2.5 and smoker prevalence does not appear to play an important role for the analysis. Concretely, it could be argued that they are positively linked to the degree of development and that they do not pose a great risk in developing countries in comparison to other risk factors. For example, while smoker prevalence was around 20% on average in Class A-2016 countries, it roughly reached 12% on average in Class C-2016 countries during the reference period. In the case of the mean annual exposure to PM2.5 there are not significant differences between the three country groups (38 μg/m^3^ on average for Class A-2016, 32 μg/m^3^ for Class B-2016 and 37 μg/m^3^ for Class C-2016). Regarding the axis defined by the second component (mainly related to HIV), the percentage of smokers and mortality due to tuberculosis showed the higher projected values (0.25 and 0.28, respectively). Curiously enough, in sub-Saharan Africa having HIV has been associated with a greater likelihood of smoking (Murphy et al., 2019), whereas tuberculosis case fatality rate is closely linked to HIV prevalence (e.g. Mukadi et al., 2001).

### 3.5 Behavioural patterns

**Fig. 6:**
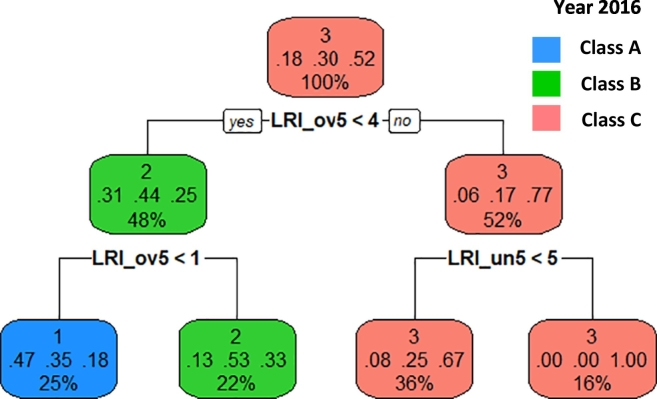
Classification decision tree regarding the number of WASH/air pollution variables correlated to LRI mortality rates. Accuracy = 69%.

Fig. 6 shows the classification decision tree for the number of WASH/air pollution variables correlated to LRI mortality rates (both over and under 5 years). Accuracy is calculated through a confusion matrix (a cross-tabulation of observed and predicted classes with associated statistics), considering a 95% confidence interval.

Therefore, in Class A-2016 African countries, the LRI mortality rate of the population over 5 years appears to be almost decoupled of WASH/air pollution variables due to improved household conditions and the causes of mortality would need to be investigated in relation to other variables. However, LRI mortality rate in children under 5 years seems to be still linked to WASH/air pollution variables in almost all African countries, although this relationship is generally stronger in the most vulnerable countries (Class C-2016).

### 3.6 Correlation and multicollinearity assessment

The computation of the correlation matrix (Table 4 in Supplementary material) shows that most of the variables are highly correlated. To group the variables according to their correlations, the dissimilarity matrix was used as input for a hierarchical clustering algorithm. Concretely, the variables were included in 6 different groups: 1) population density (Pop_dens); 2) urban population (Pop_urb), life expectancy (Life_expect), health expenditure (Health_exp), HAQ Index (HAQ_index), access to basic drinking, sanitation and hygiene services (Basic_drink, Basic_sanit, Basic_hyg), GDP per capita (GDP_cap) and mean population age (Pop_age); 3) urban agglomeration of more than 1 million inhabitants (Urb_aglom_1M); 4) total mortality rate (Mort_rate), mortality due to LRI (Mort_LRI), mortality due to LRI in children under 5 years old (LRI_un5); 5) migrant remittances per capita (Remit_cap) and ODA per capita (ODA_cap); 6) Population with HIV (Pop_HIV).

To detect potential multicollinearity issues, we computed the VIF for each variable using the mortality rate due to LRI as dependent variable and the rest of variables as explanatory ones. According to our results, three variables had a VIF more than or near 10 (Life_expect, HAQ index and Mort_rate), two variables had a VIF between 5 and 10 (Pop_age and LRI_un5), and four variables had a VIF more than 4 (Pop_urb, Health_exp, Basic_sanit and Basic_drink) (Table 5 in Supplementary Material). We decided to remove the two variables that belonged to the same group of the dependent variable (Mort_rate and LRI_un5), the ones that had the highest VIF values (Life_expect and HAQ index) and the ones that were clearly correlated with a variable which remained (Basic_sanit and Basic_drink because they were the explanatory variables of Basic_hyg in a linear regression model). After dropping these variables, Health_exp_cap and Pop_age showed similar VIF values. We decided to keep Pop_age because Health_exp_cap was highly correlated to GDP_cap. Besides, we removed the variable Urb_aglom_1M because its factor loading within the first component of the PCA (the one related to development and mortality) was below |0.6| both at continental and regional level and therefore we did not consider it as relevant for our analysis.

### 3.7 Bayesian network

**Fig. 7:**
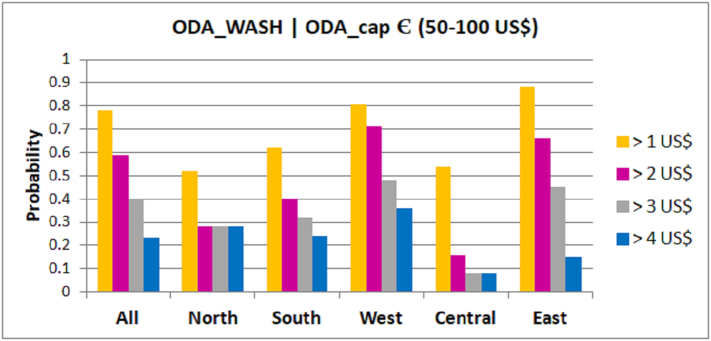
Probability of ODA per capita for the WASH sector conditioned to total ODA per capita.

Variables were discretized in three different classes (named “Low”, “Moderate” and “High”) considering equal frequency intervals (Table 6 in Supplementary material). To select the most suitable structure for the BN, several performance indicators (Log-Likelihood (LogLik), Akaike Information Criterion (AIC), Bayesian Information Criterion (BIC), Bayesian Dirichlet Equivalent score (BDE) and the logarithm of the K2 score (K2)) were compared for each of the two structure learning outcomes (Table 7 and Fig. 4 in Supplementary material). Concretely, the selected network structure (Fig. 7, Fig. 3b in Supplementary material) has three conditionally independent variables (ODA per capita, remittances per capita and GDP per capita) and it could be interpreted as follows: the access to WASH services (represented by the variable “Basic_hyg “) depends on financing sources (GDP (indirectly, through the percentage of urban population), remittances and ODA directed to WASH per capita). Urban population is related to WASH services because they generally tend to be more developed in urban areas than in rural ones. Besides, the access to WASH services has an impact on mortality due to LRI and population age, as the lack of these facilities is a risk factor not only in the development of LRI but it is also associated to other prevalent infectious diseases. Regarding other variables, the population affected by HIV also seems to influence the mortality rate due to LRI, whereas population density could be related to population age but it is not connected to LRI mortality.

According to our results, if the population access to basic hygiene services is low (less than 10% of the total population), the LRI mortality rate has a probability of 34% to be high (more than 0.12% with regard to the total population). However, if the population access to basic hygiene services is high (more than 38%), the probability of high LRI mortality significantly decreases (11%) (Table 6 in Supplementary material).

If a regional analysis is performed (splitting the data according to the African regions and fitting the selected Bayesian network to each group of data), it is possible to identify different behaviours across the continent. For example, Fig. 7 shows the probability of directing several amounts of gross ODA per capita to the WASH sector when the total net ODA per capita is between 50 and 100 US$. In Western and Eastern Africa, the probability of directing more than 1 US$ per capita to the WASH sector is more than 80%, whereas in Southern Africa decreases to 62% and in Central Africa to 54%. Moreover, the probability of directing more than 3 US$ per capita to the WASH sector is 48% in Western Africa, followed by Eastern Africa (45%), Southern Africa (32%), Northern Africa (28%) and Central Africa (8%).

### 3.8 Probabilistic reasoning

The effects of including and propagating new evidence through the BN were analyzed in four different scenarios for each cluster of data (A, B and C), using the same network structure but continuous variables:

– Scenario 1: new evidence is a value of the remittances per capita (Remit_cap) less than the first quartile (Q1) of this variable (estimated for each group). Query: what would happen if remittances are low?
– Scenario 2: new evidence is a value of the ODA per capita for the WASH sector (ODA_WASH) less than the first quartile (Q1) of this variable (estimated for each group). Query: what would happen if the ODA directed to WASH is low?
– Scenario 3: new evidence is a value of Remit_cap less than Q1 and a value of ODA_WASH more than the third quartile (Q3). Query: what would happen if the remittances are very low but the ODA directed to WASH is high?
– Scenario 4: new evidence is a value of Remit_cap more than Q3 and a value of ODA_WASH less than Q1. Query: what would happen if the remittances are high but the ODA directed to WASH is low?

**Fig. 8:**
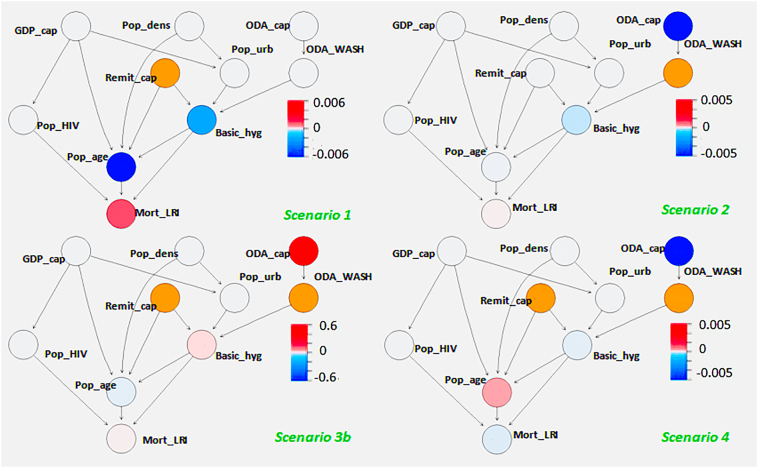
Changes in marginal distributions (cluster C).

Fig. 8 represents the changes in the marginal distributions of the variables for the cluster C (high vulnerability) in the three scenarios. Yellow nodes are the ones where new evidence is included, red nodes are those where the mean of the marginal distribution increases and blue ones where this mean decreases (intense colours correspond to pronounced changes).

In Scenario 1, the new evidence (Remit_cap = 3 US $) causes a decrease in WASH access (represented by Basic_hyg), a slight increase in LRI mortality (Mort_LRI) and a decrease in population age (Pop_age). In Scenario 2, assigning a value of ODA_WASH = 0.5 US $ decreases the mean of the marginal distribution for WASH access (less than in Scenario 1), although it practically does not affect the rest of the network. In Scenario 3a, the new evidence (Remit_cap = 3 US $ and ODA_WASH = 5 US $) causes similar changes to the ones in Scenario 1, but of lesser magnitude because the value assigned to ODA_WASH somehow compensates the low remittance value. Furthermore, in Scenario 3b (Remit_cap = 3 US $ and ODA_WASH = 15 US $), the higher value of ODA_WASH is able to increase the mean of the marginal distribution of WASH access, although mortality due to LRI practically remains unaffected and population age still decreases (less than in Scenario 1). Finally, in Scenario 4 (Remit_cap = 30 US $ and ODA_WASH = 0.5 US $), the results are very similar to the baseline scenario, although both the access to basic hygiene and the LRI mortality rate experiment an slight decrease. Therefore, the larger perturbation in the network happens when remittances are low (Scenario 1). A possible explanation could be that the low value of remittances would entail a reduction of the health expenditure at the household level, thereby affecting negatively to the well-being of the population.

However, different results emerge when the same analysis is performed for the two additional clusters. Cluster B (intermediate vulnerability) shows the same behaviour of cluster C in Scenario 1 (a low value assigned to remittances decreases WASH access and increases LRI mortality); however, the role of ODA directed to WASH is not clear: the higher the value assigned to this variable, the lower the access to WASH. Besides, a high value of remittances seems to have a positive effect, improving the access to basic hygiene services and reducing LRI mortality. In the case of cluster A (low vulnerability), neither migrant remittances nor ODA directed to WASH seems to be related to WASH access, as both a lower value of remittances or ODA causes an increase in access to WASH, whereas high values assigned to these variables decrease it (Tables 8 and 9 in Supplementary material).

### 3.9 Insights with regard to COVID-19

For each of the 21 African countries where daily data on number of tests, confirmed cases and deaths exist, we computed the average of the daily ratios of confirmed cases/number of tests and deaths/confirmed cases. Here, it should be noted that these countries often lack continuous data on these variables, thus we preferred to use the daily smoothed variables provided in the dataset (calculated as the seven-day moving average over a complete, linearly interpolated daily series).

As expected, we observed that countries belonging to cluster A have better testing capacities (in Cabo Verde and Morocco, the daily average of tested population is 0.07% and 0.04%). Besides, cluster B countries tested daily an average of 0.04% of the total population (South Africa, Namibia). However, cluster C countries lag clearly behind (e.g. 0.006% in Uganda, Ethiopia or Côte d'Ivoire). Regarding daily rates of deaths/confirmed cases, a clear trend does not emerge with regard to the three country groups, although it should be noted that some countries with really low testing capacities have a high rate of confirmed cases (such as the Democratic Republic of the Congo, where the daily average of tested population is 0.0004% and the daily ratio of confirmed cases/number of tests is around 20%) (Table 10 in Supplementary material). Therefore, this could indicate a severe underreporting problem (Hasell et al., 2020).

Regarding the potential correlations among the average daily rates and the variables considered in the current study, in general they show low values and further research should be conducted on this issue. The ratio of confirmed cases/number of tests is mainly correlated to the percentage of urban population (0.34), the access to basic hygiene services (0.28), the GDP per capita (0.21) and the population age (0.19). Besides, the ratio of deaths/confirmed cases is mainly correlated to the percentage of population affected by HIV (0.41).

## 4 Discussion

### 4.1 Poor access to WASH services as a risk factor in LRI development

The analysis of the incidence and risk factors involved in the development of other respiratory diseases in Africa could provide valuable clues to contain the current COVID-19 pandemic in the continent. According to our results, there is a high and negative correlation between the access to basic WASH services and LRI mortality. Troeger et al. (2018) pointed out that people at highest risk for contracting or dying from lower respiratory infections often come from overcrowded households with inconsistent or insufficient access to adequate nutrition (e.g. non-exclusive breastfeeding, child underweight, wasting and stunting, vitamin A and zinc deficiency, etc.), clean cooking fuels, vaccines, and sanitation, or have immunocompromising conditions. In addition, they suggested that although there has been a substantial decrease in lower respiratory infection mortality since 1990, the majority of the remaining deaths could be avertable and increased global investment in prevention and treatment interventions is still required. Concretely, Stanaway et al. (2018) attributed 229,000 and 188,000 LRI deaths worldwide to the lack of access to handwashing facilities in years 2007 and 2017, respectively. Besides, Prüss-Ustün et al. (2019) estimated that 13% of the overall disease burden of acute respiratory infections was attributable to inadequate handwashing with soap and water (370,000 deaths worldwide in 2016, 134,000 in Sub-Saharan Africa).

### 4.2 ODA and enhanced access to WASH services

Since 2000, the overall capacity of Africa to face up LRI has improved according to the combined vulnerability index proposed in this study. However, more than a half of African countries could still be included within the most vulnerable group of the three identified ones. Our findings suggest that these countries are still highly dependent on ODA to develop their WASH facilities. However, there are significant differences in the distribution of ODA across the continent. While Western Africa received more than 4 US$ per capita during the period 2003–2011, in Central and Southern Africa was significantly lower (2.2 and 2.6 US$, respectively). According to Cha et al. (2017), the inequality of water and sanitation coverage among countries across the world has not been addressed effectively during the past decade. In addition, they showed that the countries with the least coverage persistently received far less ODA per capita than did countries with much more extensive water and sanitation coverage, suggesting that ODA for water and sanitation is poorly targeted. According to these authors, 75% of pneumonia and diarrhoea-specific child deaths take place in 15 countries around the globe, and 9 of them are in Africa (Angola, Burkina Faso, DR Congo, Kenya, Mali, Niger, Nigeria, Tanzania and Uganda). However, none of these countries had more than US$ 100 per capita committed for WASH during the period 2000–2010 (e.g. Burkina Faso (US$ 48.59), Tanzania (US$ 32.63), Kenya (US$ 26.30); Uganda (US$ 25.58); Niger (US$ 23.62); Angola (US$ 16.39); Malawi (US$ 12.67); Ethiopia (US$ 12.23) and Nigeria (US$ 7.01)) (Cha et al., 2017).

Historically, the water sector has attracted smaller amounts of ODA than other social sectors, including education, health, population planning, and government and civil society. Although the ODA directed to the water sector increased by 90% during the period 1995–2014 (rising from US$6.8 billion to $12.9 billion per year, in constant 2014 prices), overall ODA increased by more than 230%. During this 20-year period, sub-Saharan Africa was one of the largest recipients worldwide of ODA for the water sector (25% of the total) (Winpenny et al., 2016). However, the Eastern and Southern and the West and Central Africa regions still lag behind the rest of the world in terms of access to at least basic sanitation coverage, and significant disparities can be seen in rural and urban coverage for both water and sanitation (Jones et al., 2019). In addition, these authors found that foreign funds devoted to the WASH sector have undergone a significant reduction since 2012, with foreign aid dropping from 60% to less than 50% of the total. The main driver behind the fall was the reduction in overall capital allocations by sub-Saharan African countries in the sample, from 1% of GDP in 2012 to less than 0.5% in 2015. However, this situation seems to have improved in recent years: ODA commitments for WASH increased during the period 2015–2017, almost doubling in sub-Saharan Africa (WHO and UN-Water, 2019). Although the relationship between ODA directed to the WASH sector and increased access to these services is non-linear in sub-Saharan Africa (Ndikumana and Pickbourn, 2017), its role in the achievement of priorities' development goals cannot be disregarded due to the cross-cutting nature of SDGs (UN General Assembly, 2015).

### 4.3 Perspectives on the role of remittances on healthier households

The latest UN-Water GLAAS report on hygiene acknowledges that household expenditures on these services are high compared to the governmental ones (WHO and UN-Water, 2020). In this regard, our results also suggest that remittances are playing a significant role in the access to healthcare and WASH services and poverty reduction, as pointed out by Mohapatra and Ratha (2011), Rodima-Taylor (2015) or Hagen-Zanker (2015). Remittances have the advantage of generally being well targeted to the needs of their recipients, and many studies show a positive correlation between remittances and health improvement at the household level, which is coherent with our results. For example, McKenzie and Hildebrandt (2005) concluded that migration raises health knowledge in addition to the direct effect on wealth, after examining the impact of remittances on children in rural Mexico. However, it is also to be pointed out that migration will not always have positive, poverty-reducing impacts on a household-level. For example, migrant-sending households often take up huge loans to finance migration and these can take years to repay, at often large interest rates. In addition, while spending on food and other basic needs is a top priority for households, there are few studies that show migrants are more likely to spend remittances on unproductive or ‘status-oriented’ consumption (Hagen-Zanker, 2015).

For many developing countries, remittances constitute a large source of foreign income relative to other financial flows. Between 1995 and 2010, remittances to sub-Saharan Africa increased by 545% and reached $18 billion in 2010 (UNDP, 2011), representing almost 60% of the size of official aid flows in the region (Mohapatra and Ratha, 2011). In any case, remittances serve as a lifeline for many African countries, and some of these countries rely heavily on them (such as Lesotho, where the remittances received were up to 25% of the GDP in the period 2004–2018) (Sayeh and Chami, 2020). Recently, these authors have pointed out that the pandemic will deliver a blow to remittance flows that may be even worse than during the financial crisis of 2008 (decreasing by about $100 billion in 2020). Concretely, in sub-Saharan Africa the World Bank estimated a decline of remittances by 23.1% in 2020 (World Bank, 2020), due to the possibility of migrant workers losing their jobs and returning to unemployment in their countries of origin. However, Bisong (2020) pointed out large differences across all African countries: although international remittances have kept flowing into some households (due to labour laws and extensive welfare packages in North American and European migrant-host countries), intra-African remittances have generally decreased. Besides, a proper tracking of African remittances within countries could be crucial, as internal remittances are largely unknown in comparison with the international ones and could be threatened to a great extent in the current pandemic context. This is particularly important when it comes to the poorest households in rural areas, where internal remittances sent from urban areas often represent the difference between subsistence and food insecurity (Adhikari, 2020). In this context, donor countries and international financial institutions should be urged to help migrant-source countries not only fight the pandemic, but also to cushion the shock of losing these private income flows (Sayeh and Chami, 2020).

### 4.4 Potential limitations and future perspectives

In any case, it is necessary to keep in mind that the approach selected in this study is limited by its sectoral nature, while the COVID-19 pandemic is a highly multidimensional problem. Aspects that have not been considered in this research, such as genetic factors, climate characteristics, vaccination against tuberculosis, political instability or extension of transportation networks could play an important role in the current context. Besides, it should be noted that we could be on the verge of the next zoonotic outbreak due the degradation of natural environments (Córdoba-Aguilar et al., 2021). Moreover, LRI mortality has been used as a proxy variable to study the potential links between COVID-19 and other variables, but its behaviour could differ in relation to other respiratory diseases. In fact, while mortality due to COVID-19 is higher among elderly people than in other age groups, LRI mortality in developing countries is prevalent in children under 5 years old. Although the pandemic was expected to be devastating in Africa (due to high poverty levels, weak health systems, overpopulation and poor hygienic practices), the reported case fatality has remained significantly low in the continent (WHO, 2020a). Nevertheless, the lower rate of transmission could result in a long-lasting outbreak of several years, spreading at a slow but steady pace from hotspots to other areas (WHO, 2020b). Besides, multiple authors point out that the real incidence of the pandemic in Africa has been widely underestimated (Moultrie et al., 2021; Mwananyanda et al., 2021; Watson et al., 2020). Therefore, this is not the time to let down the guard: future waves in Africa could be more and more difficult to restrain due to lack of preparedness (Ghosh et al., 2020) and mortality rates related to other prevalent diseases could rise (e.g. the elderly might may not risk to take children for malaria medication if they are scared of contracting COVID-19, hence increasing malaria-related mortalities among children (Nghochuzie et al., 2020)). Besides, lack of proper wastewater treatment not only could favour the faecal-oral transmission of SARS-CoV-2 (e.g. Gwenzi, 2021; Guo et al., 2021; Arslan et al., 2020) but also contribute to the development of antibiotic resistance (due to continuous contact with faecal matter), which could hamper the ability to treat all kinds of infectious diseases (potentially including coronaviruses like SARS-CoV-2) (Graham and Collignon, 2020). Finally, it should be highlighted that local COVID-19 research is urgently needed in Africa to address knowledge gaps and formulate appropriate policy and decision making (Gwenzi and Rzymski, 2021).

## 5 Conclusions

The present study aims to explore the evidence-based link between the development of respiratory infections and the access to WASH services in Africa, as well as the role of other socioeconomic variables such as financial sources or demographic factors, in order to provide scientific insights that could guide the integration of enhanced WASH services within the international response to the current COVID-19 pandemic. This strategy will not only allow to curb the spread of the SARS-CoV-2 through the implementation of highly recommended preventive measures (such as handwashing), but will also contribute to fight other prevalent infectious diseases that could eventually rise if the response to COVID-19 neglects them.

Key findings include that the estimated vulnerability of the continent to LRI has decreased during the period 2000–2016, although still more than half of the African countries could be included within the most vulnerable group (Class C-2016, characterized by weak healthcare systems, low access to WASH services and high LRI mortality rates). Besides, LRI mortality in people over 5 years old and poor WASH access/air pollution only seems to be decoupled in the less vulnerable African countries (Class A-2016), whereas these variables show a strong relationship regarding the population segment under 5 years old for all the country groups. According to our results, the probability of having a high LRI mortality rate (more than 0.12%) is 0.34 when the access to basic hygiene services is low (less than 10%), whereas it decreases to 0.11 when the access to basic hygiene is more than 38%.

With regard to financing sources directed to the WASH sector, our findings suggest that the contribution of ODA is heterogeneous across the continent (being Western Africa a main recipient region). Besides, migrant remittances seems to play a key role in health and home improvement at the household level, including an enhanced access to WASH services. In this context, the expected decrease of migrant remittances due to the pandemic could have an important impact on the well-being of population living in the most vulnerable African countries, which could be alleviated to some extent through properly targeted ODA.

Finally, little evidence could be extracted from the analysis of COVID-19 data in the continent, although the daily share of positive cases could be correlated with the percentage of urban population, the access to basic hygiene services, the GDP per capita and the population age, while the daily ratio of deaths in relation to confirmed cases seems to be related to the percentage of population affected by HIV. Despite these relationships could be potentially plausible, they are highly speculative due to the reduced availability of reliable data and therefore further research should be conducted on these issues.

## CRediT authorship contribution statement

**P. Marcos-Garcia:** Conceptualization, Methodology, Software, Visualization, Writing – original draft. **C. Carmona-Moreno:** Conceptualization, Methodology, Supervision, Writing-review and editing. **J. López-Puga:** Supervision. **A.M. Ruiz-Ruano García:** Supervision.

## Declaration of competing interest

The authors declare that they have no known competing financial interests or personal relationships that could have appeared to influence the work reported in this paper.

## Appendix A Supplementary data

Supplementary material Image 1
