# Supplementary material for: COVID-19 pandemic in Africa: Is it time for water, sanitation and hygiene to climb up the ladder of global priorities?
Source: Sci Total Environ. 2021 Oct 15;791:148252. doi: 10.1016/j.scitotenv.2021.148252 (PMC8173594; doi:10.1016/j.scitotenv.2021.148252)
Supplement: Supplementary file 1 — Supplementary material [file mmc1.docx]

**SUPPLEMENTARY MATERIAL**

**Tables**

*Table 1. Description of selected variables*

| Variable | Units | Short ID | Main source |
| --- | --- | --- | --- |
| Population | 10^3^ people | Population | UN |
| Population density | People/km^2^ | Pop_dens | World Bank |
| Urban population | % total population | Pop_urb | World Bank |
| Population living in big cities | % total population | Urb_aglom_1M | World Bank |
| Median age of the total population | Years | Pop_age | UN |
| Life expectancy at birth | Years | Life_expect | World Bank |
| Health expenditure | % GDP | Health_exp | World Bank |
| Healthcare Access and Quality Index | Dimensionless (0-100) | HAQ_index | IHME |
| Gross Domestic Product (GDP) | 10^6^ current US$ | GDP | World Bank |
| Migrant remittance inflows | 10^6^ current US$ | Remit | World Bank |
| Net official development assistance | 10^6^ current US$ | ODA | World Bank |
| Gross official development assistance for the WASH sector | 10^6^ current US$ | ODA_WASH | World Bank |
| Total deaths | Number of deaths | Total_deaths | WHO |
| Lower respiratory infection (LRI) deaths | Number of deaths | LRI_deaths | WHO |
| LRI deaths in children under 5 years | % LRI deaths | LRI_un5 | WHO |
| Malaria deaths | Number of deaths | Malaria | WHO |
| Tuberculosis deaths | Number of deaths | Tuberculosis | WHO |
| Diarrhoea deaths | Number of deaths | Diarrhoea | WHO |
| Schistosomiasis deaths | Number of deaths | Schistosomiasis | WHO |
| Protein-energy malnutrition deaths | Number of deaths | PE_Malnut | WHO |
| People living with HIV | Number of people | HIV_infected | WHO |
| Access to basic drinking water services | % total population | Basic_drink | WHO/UNICEF |
| Access to basic sanitation services | % total population | Basic_sanit | WHO/UNICEF |
| Access to basic handwashing services | % total population | Basic_hyg | WHO/UNICEF |
| Access to clean cooking fuels | % total population | Clean_cooking | World Bank |
| Smoker prevalence | % total population | Smokers | World Bank/IHME |
| Exposure to PM2.5 levels over Tier 1 | % total population | PM2.5_T1 | World Bank |
| Exposure to PM2.5 levels over Tier 2 | % total population | PM2.5_T2 | World Bank |
| Mean annual exposure to PM2.5 | μg/m^3^ | PM2.5_MA | World Bank |
| Domestic water withdrawal | m^3^/ year | Dom_withdr | Huang et al. (2018) |

*Table 2. Subsets of variables.*

| Set_1_ | Set_2_ | Set_3_ | Set_4_ |
| --- | --- | --- | --- |
| Pop_urb | Pop_urb | Basic_sanit | Clean_cooking |
| Life_expect | Life_expect | Basic_drink | Smokers |
| Basic_sanit | Basic_sanit | Basic_hyg | PM2.5_T1 |
| GDP_cap | GDP_cap | Clean_cooking | PM2.5_T2 |
| Remit_cap | Remit_cap | Smokers | PM2.5_MA |
| ODA_cap | ODA_cap | PM2.5_T1 | Malaria |
| Pop_age | Pop_age | PM2.5_T2 | Tuberculosis |
| Mort_LRI | Mort_LRI | PM2.5_MA | Diarrhoea |
| LRI_un5 | LRI_un5 | LRI_un5 | Schistosomiasis |
| Population |  | Mort_LRI | PE_Malnut |
| Pop_dens |  |  |  |
| Urb_aglom_1M |  |  |  |
| HAQ_index |  |  |  |
| Health_exp |  |  |  |
| Mort_total |  |  |  |
| Basic_drink |  |  |  |
| Basic_hyg |  |  |  |
| Pop_HIV |  |  |  |

*Table 3. First component of the PCA at both continental and regional levels. Amount of variance explained: All (58%), East(56%), Central (49%), South (58%), West (54%) and North (59%).*

|  | **All** | **East** | **Central** | **South** | **West** | **North** |
| --- | --- | --- | --- | --- | --- | --- |
| **Pop_dens** | 0.28 | 0.74 | 0.49 | -0.66 | 0.51 | 0.36 |
| **Pop_urb** | 0.64 | 0.40 | 0.92 | 0.95 | 0.82 | 0.83 |
| **Urb_aglom_1M** | 0.05 | -0.53 | -0.14 | 0.54 | -0.11 | 0.18 |
| **Life_expect** | 0.81 | 0.84 | 0.83 | 0.92 | 0.80 | 0.96 |
| **Health_exp_cap** | 0.71 | 0.87 | 0.75 | 0.82 | 0.86 | 0.71 |
| **HAQ_index** | 0.93 | 0.92 | 0.88 | 0.95 | 0.88 | 0.95 |
| **Mort_rate** | -0.73 | -0.61 | -0.79 | -0.79 | -0.77 | -0.81 |
| **Basic_sanit** | 0.86 | 0.85 | 0.65 | 0.65 | 0.78 | 0.92 |
| **Basic_drink** | 0.86 | 0.87 | 0.87 | 0.93 | 0.81 | 0.89 |
| **Basic_hyg** | 0.80 | 0.72 | 0.74 | 0.88 | 0.48 | 0.86 |
| **GDP_cap** | 0.61 | 0.91 | 0.58 | 0.88 | 0.86 | 0.67 |
| **Remit_cap** | 0.61 | 0.71 | 0.54 | -0.41 | 0.89 | 0.49 |
| **ODA_cap** | 0.20 | 0.32 | 0.39 | -0.07 | 0.71 | -0.08 |
| **Pop_age** | 0.89 | 0.95 | 0.77 | 0.69 | 0.81 | 0.90 |
| **Pop_HIV** | 0.01 | -0.30 | 0.26 | -0.17 | -0.20 | -0.76 |
| **Mort_ LRI** | -0.81 | -0.71 | -0.79 | -0.89 | -0.73 | -0.92 |
| **LRI_un5** | -0.86 | -0.95 | -0.88 | -0.95 | -0.80 | -0.95 |

*Table 4. Correlation matrix (Set_1_)*

*
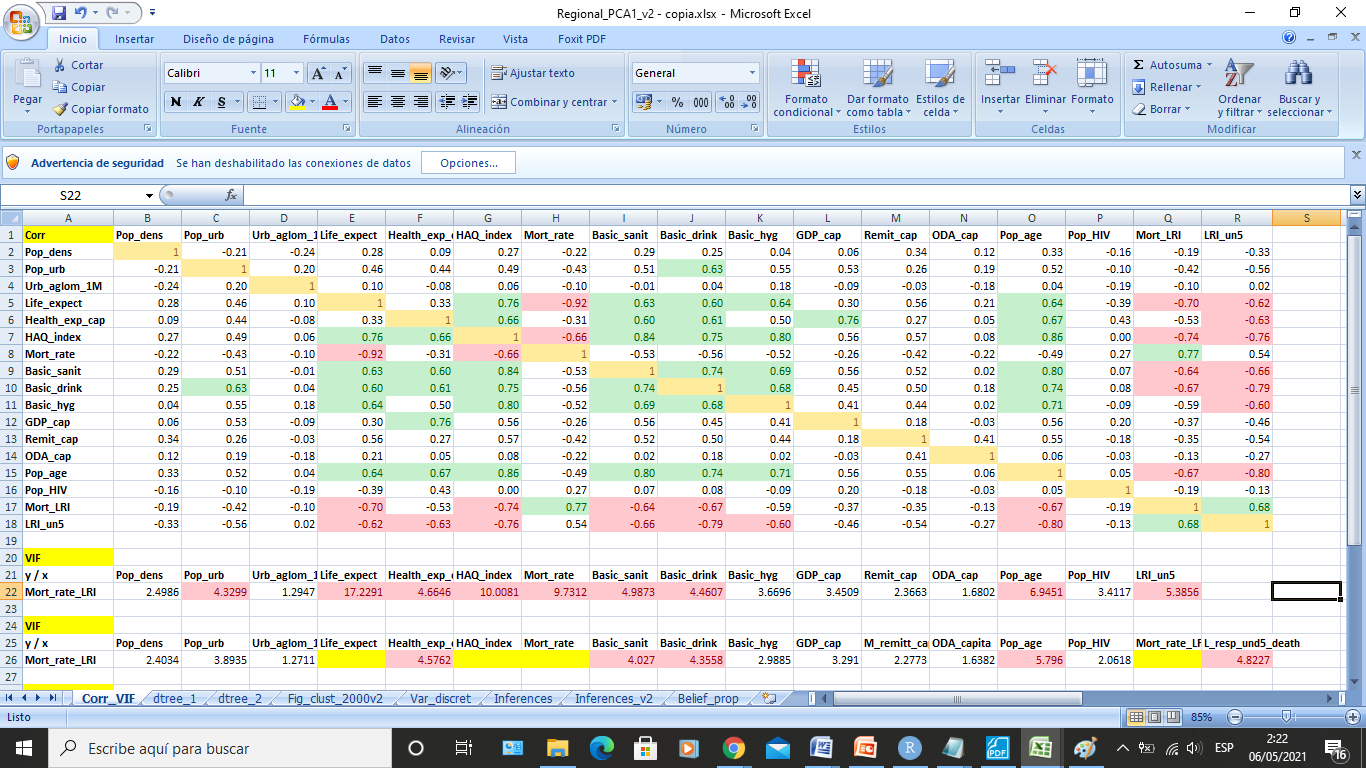
*

*Table 5. Initial VIF values (if the dependent variable is the mortality rate due to LRI) and after variable selection*

*
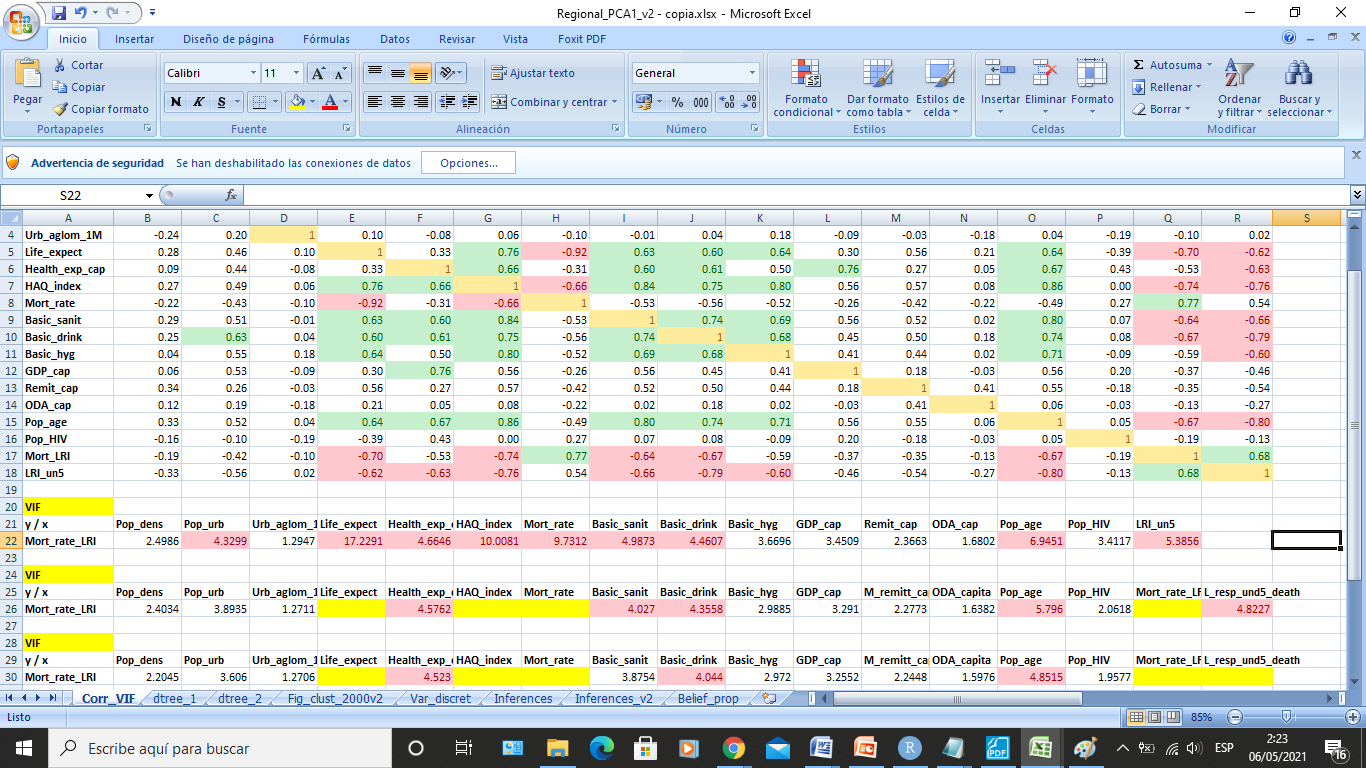
*

*
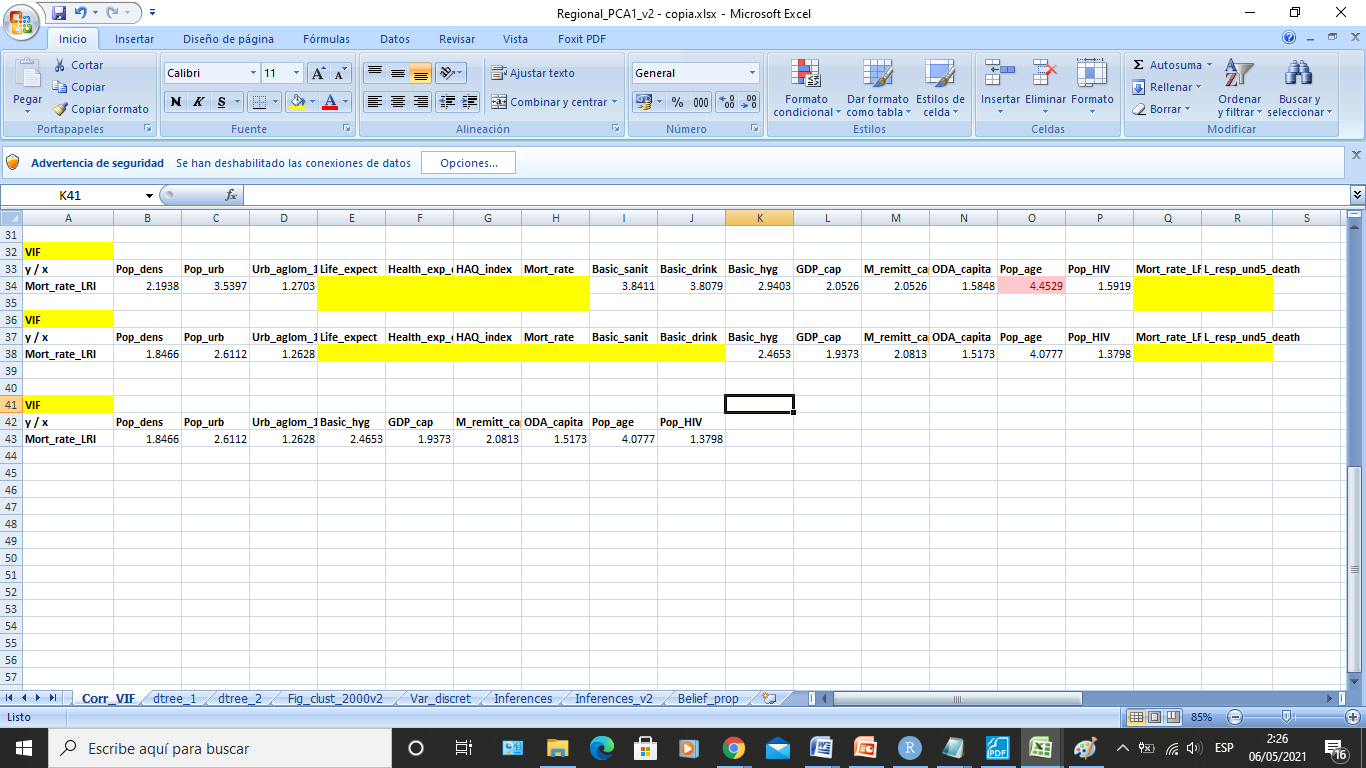
*

*Table 6. Variable discretization using equal frequency intervals*

| **Variable** | **Units** | **Low** | **Moderate** | **High** |
| --- | --- | --- | --- | --- |
| **Pop_dens** |  | <33 | 32.5-72 | >72 |
| **Pop_urb** | (%) | <33 | 33-46 | >46 |
| **Basic_hyg** | (%) | <10 | 10-38 | >38 |
| **GDP_cap** | (US$) | <512 | 512-1315 | >1315 |
| **Remit_cap** | (US$) | <7 | 7-33 | >33 |
| **ODA_cap** | (US$) | <38 | 38-67 | >67 |
| **ODA_WASH_cap** | (US$) | <1 | 1-3 | >3 |
| **Pop_age** | (years) | <17.5 | 17.5-19 | >19 |
| **Pop_HIV** | (%) | <0.88 | 0.88-2.35 | >2.35 |
| **Mort_ LRI** | (%) | 0.012-0.09 | 0.09-0.12 | 0.12-0.27 |

*Table 5. Scores of each bayesian network structure regarding the discretized variables*

|  | BIC | AIC | BDE | K2 | LogLik |
| --- | --- | --- | --- | --- | --- |
| Structure a) | -12628.28 | -6523.907 | -4432.153 | -4169.37 | -3503.907 |
| Structure b) | -4726.424 | -4103.859 | -4432.552 | -4173.567 | -3795.859 |

*Table 6. Conditional probability table for LRI mortality regarding the access to basic sanitation services*

|  | **Access to basic hygiene services** | | |
| --- | --- | --- | --- |
| **LRI mortality** | **Low** | **Moderate** | **High** |
| **Low** | 0.03 | 0.12 | 0.43 |
| **Moderate** | 0.26 | 0.24 | 0.16 |
| **High** | 0.34 | 0.28 | 0.11 |

*Table 7. Mean of the marginal distributions for cluster C scenarios. Scenario 1 (Remit_cap=3), Scenario 2 (ODA_WASH=0.5), Scenario 3a (Remit_cap=3 and ODA_WASH=5), Scenario 3b (Remit_cap=3 and ODA_WASH=15) and Scenario 4 (Remit_cap=30 and ODA_WASH=0.5)*

|  | Baseline | Scenario_1 | Scenario_2 | Scenario_3a | Scenario_3b | Scenario_4 |
| --- | --- | --- | --- | --- | --- | --- |
| Basic_hyg | 16.7059975 | 15.774754 | 16.4015520 | 16.3474018 | 18.2964981 | 16.6758097 |
| Mort_LRI | 0.1296943 | 0.131906 | 0.1298157 | 0.1316777 | 0.1309006 | 0.1291643 |

*Table 8. Mean of the marginal distributions for cluster B scenarios. Scenario 1 (Remit_cap=9), Scenario 2 (ODA_WASH=0.5), Scenario 3a (Remit_cap=9 and ODA_WASH=7), Scenario 3b (Remit_cap=9 and ODA_WASH=10) and Scenario 4 (Remit_cap=40 and ODA_WASH=0.5).*

|  | Baseline | Scenario_1 | Scenario_2 | Scenario_3a | Scenario_3b | Scenario_4 |
| --- | --- | --- | --- | --- | --- | --- |
| Basic_hyg | 33.20476897 | 32.94074379 | 34.80676687 | 31.54232584 | 30.1575185 | 35.0401879 |
| Mort_LRI | 0.08611264 | 0.08618477 | 0.08423907 | 0.08782025 | 0.08943982 | 0.0841753 |

*Table 9. Mean of the marginal distributions for cluster A scenarios. Scenario 1 (Remit_cap=50), Scenario 2 (ODA_WASH=0.5), Scenario 3a (Remit_cap=50 and ODA_WASH=9), Scenario 3b (Remit_cap=50 and ODA_WASH=15) and Scenario 4 (Remit_cap=200 and ODA_WASH=0.5).*

|  | Baseline | Scenario_1 | Scenario_2 | Scenario_3a | Scenario_3b | Scenario_4 |
| --- | --- | --- | --- | --- | --- | --- |
| Basic_hyg | 66.14216852 | 84.61799376 | 65.1428093 | 85.11215805 | 86.16640991 | 58.59073432 |
| Mort_LRI | 0.03070926 | 0.02493969 | 0.0310251 | 0.02478352 | 0.02445033 | 0.03307116 |

*Table 10. Average daily ratios of deaths/confirmed cases and confirmed cases/tested.*

| **Country** | **Ratio(tested/population) (%)** | **Ratio positive/tested) (%)** | **Ratio(death/positive) (%)** |
| --- | --- | --- | --- |
| Cabo Verde | 0.0713 | 14.7043 | 0.8731 |
| Côte d'Ivoire | 0.0059 | 8.2053 | 0.7317 |
| Congo DRC | 0.0004 | 19.8087 | 2.5219 |
| Ethiopia | 0.0056 | 8.7766 | 1.5017 |
| Gambia | 0.0067 | 7.3840 | 3.7122 |
| Ghana | 0.0086 | 8.3109 | 0.9628 |
| Kenya | 0.0071 | 6.9966 | 2.4297 |
| Madagascar | 0.0017 | 15.9478 | 1.8224 |
| Malawi | 0.0031 | 8.9092 | 3.1426 |
| Mauritania | 0.0135 | 5.6622 | 1.8873 |
| Morocco | 0.0380 | 8.9659 | 1.9914 |
| Mozambique | 0.0042 | 9.5735 | 0.9252 |
| Namibia | 0.0428 | 10.4987 | 1.1034 |
| Nigeria | 0.0024 | 10.0490 | 1.4700 |
| Rwanda | 0.0259 | 1.5818 | 1.2568 |
| Senegal | 0.0071 | 7.8213 | 2.4037 |
| South Africa | 0.0428 | 11.4494 | 4.0719 |
| Togo | 0.0091 | 3.6348 | 2.4273 |
| Uganda | 0.0058 | 4.5808 | 1.0194 |
| Zambia | 0.0189 | 6.7235 | 2.6189 |
| Zimbabwe | 0.0091 | 6.2596 | 3.6314 |

**Figures**

*Figure 1. Households with a separate room for cooking depending on household type in 2009*

*
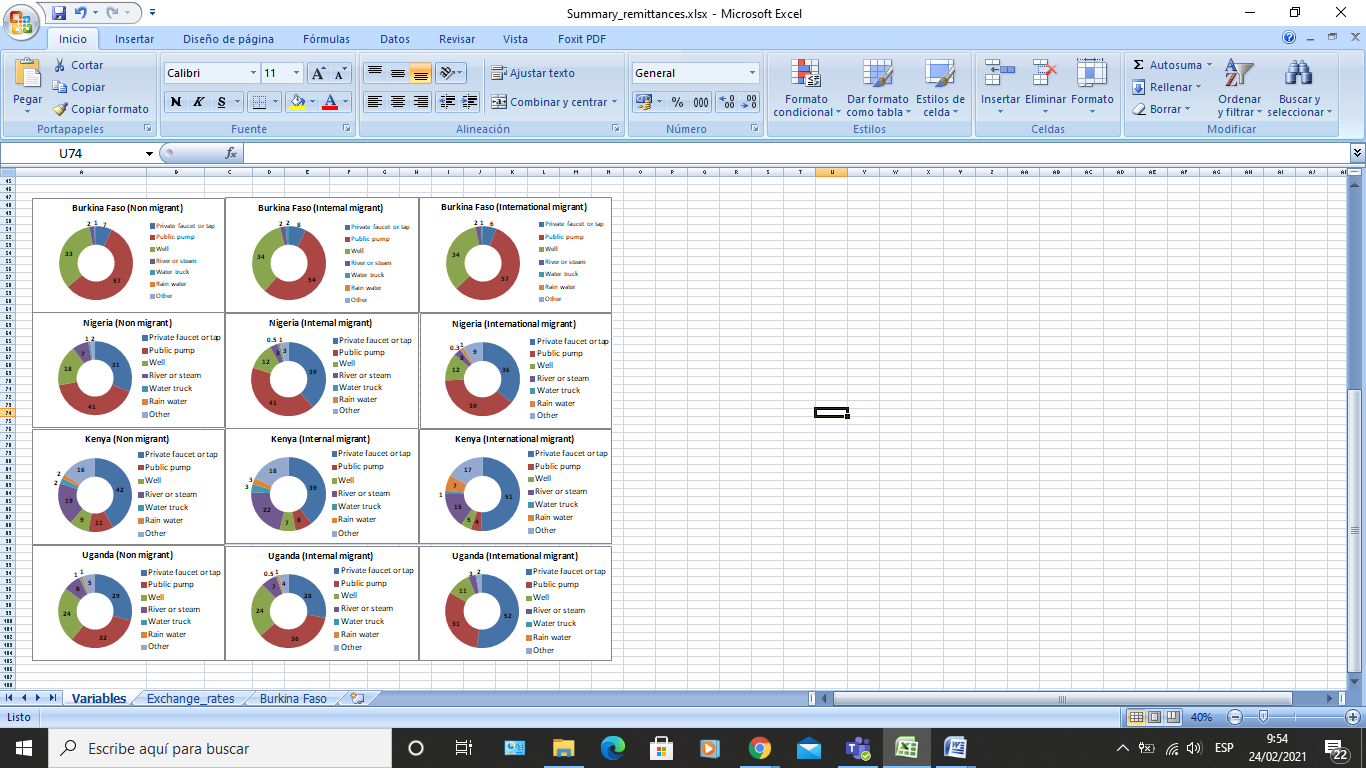
*

*Figure 2. Primary source of drinking water in each household type (non migrant, internal migrant and international migrant)*

*
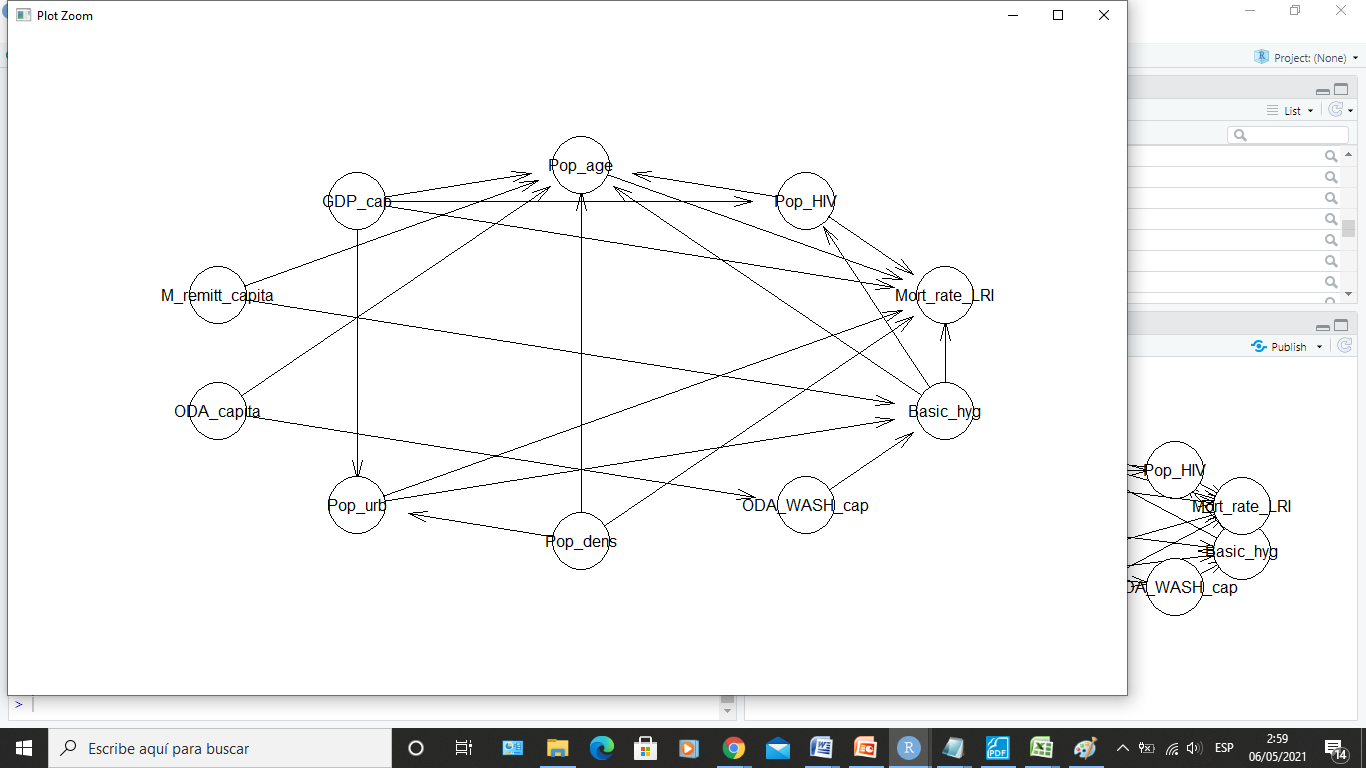
*

**a)**


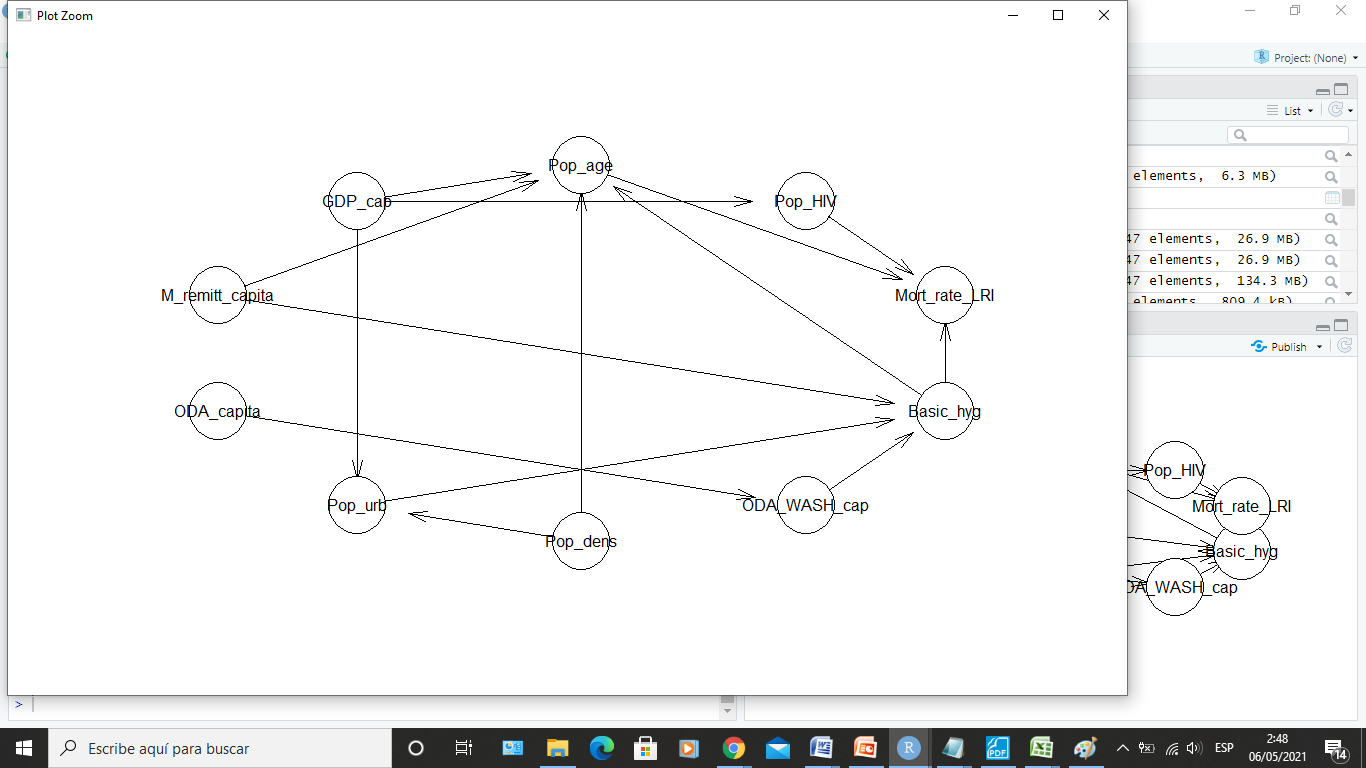


**b)**

*Figure 3. Network structures according to: a) Hill Climbing algorithm; b) Max-Min Hill Climbing algorithm.*

*
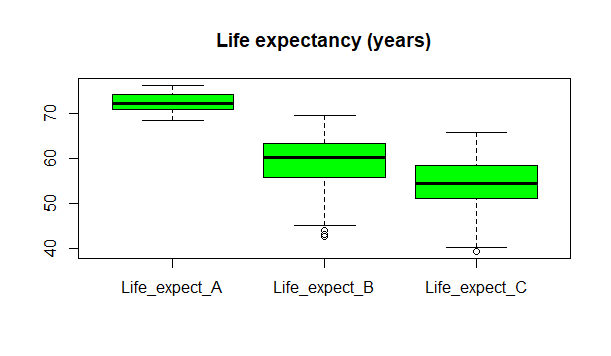
* *
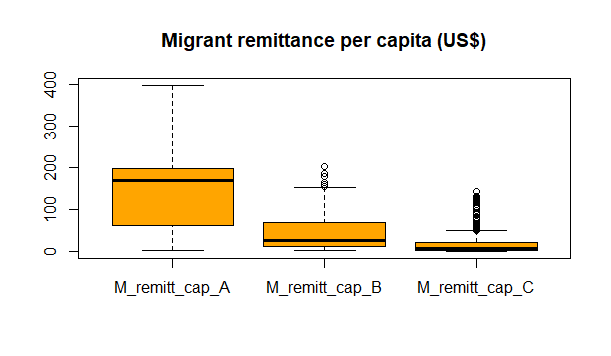
*


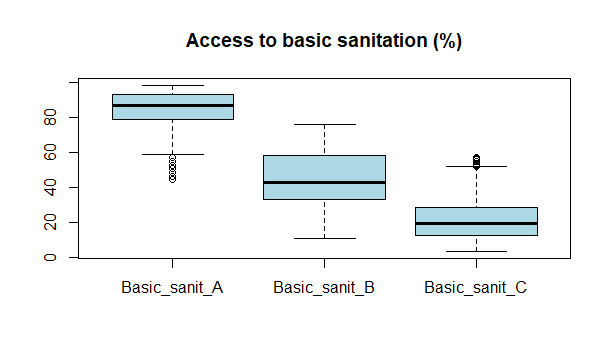
 *
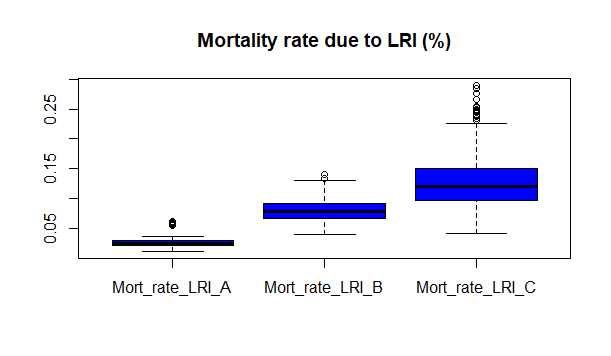
*


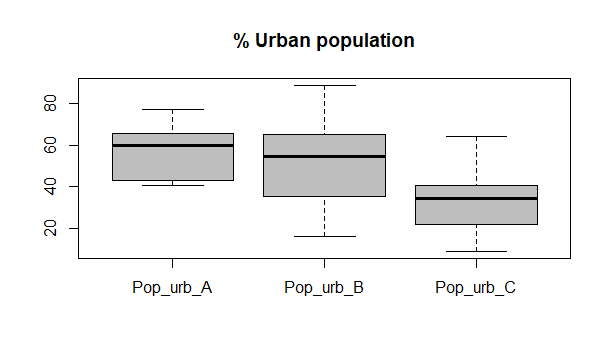

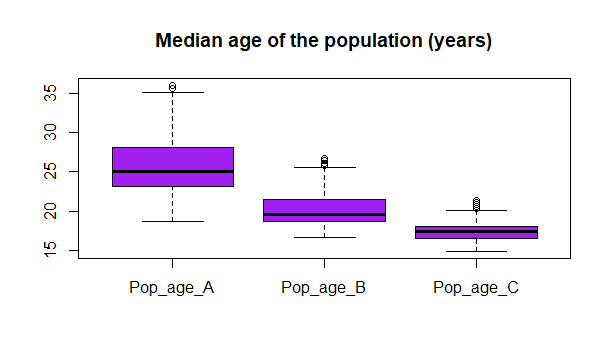

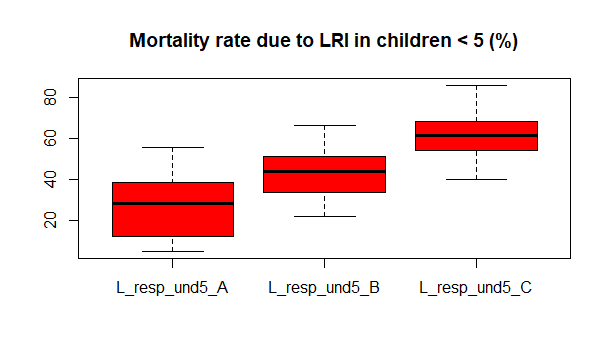

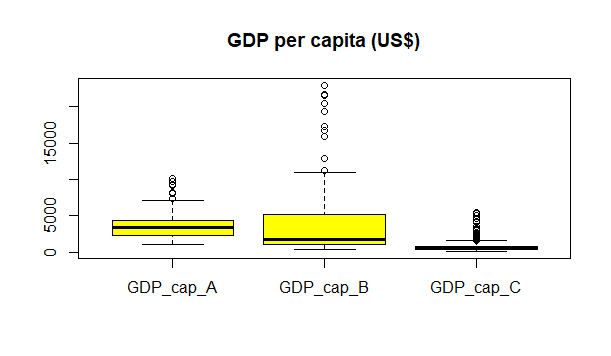


*Figure 4. Boxplots for each cluster of countries and several variables*

**MULTIVARIATE REGRESSION TO COMPLETE MISSING VALUES IN HYGIENE DATA**

**All data**

Call:

lm(formula = Basic_hyg ~ Basic_drink + Basic_sanit, data = df)

Residuals:

Min 1Q Median 3Q Max

-30.536 -9.961 0.611 8.845 45.575

Coefficients:

Estimate Std. Error t value Pr(>|t|)

(Intercept) -27.88775 3.07297 -9.075 < 2e-16 ***

Basic_drink 0.61562 0.06232 9.878 < 2e-16 ***

Basic_sanit 0.36255 0.04367 8.302 1.5e-15 ***

---

Signif. codes: 0 ‘***’ 0.001 ‘**’ 0.01 ‘*’ 0.05 ‘.’ 0.1 ‘ ’ 1

Residual standard error: 14.83 on 410 degrees of freedom

(559 observations deleted due to missingness)

Multiple R-squared: 0.5771, Adjusted R-squared: 0.5751

F-statistic: 279.8 on 2 and 410 DF, p-value: < 2.2e-16

**Algeria**

Call:

lm(formula = Basic_hyg ~ Basic_drink + Basic_sanit, data = Algeria)

Residuals:

Min 1Q Median 3Q Max

-0.052907 -0.029486 0.000889 0.011349 0.089911

Coefficients:

Estimate Std. Error t value Pr(>|t|)

(Intercept) 40.3889 8.7439 4.619 0.00362 **

Basic_drink 1.1851 1.3355 0.887 0.40904

Basic_sanit -0.7719 1.5198 -0.508 0.62963

---

Signif. codes: 0 ‘***’ 0.001 ‘**’ 0.01 ‘*’ 0.05 ‘.’ 0.1 ‘ ’ 1

Residual standard error: 0.04755 on 6 degrees of freedom

(9 observations deleted due to missingness)

Multiple R-squared: 0.9758, Adjusted R-squared: 0.9678

F-statistic: 121.1 on 2 and 6 DF, p-value: 1.414e-05

**Angola**

Call:

lm(formula = Basic_hyg ~ Basic_drink + Basic_sanit, data = Angola)

Residuals:

15 16 17 18

-7.636e-05 -1.748e-04 5.808e-04 -3.296e-04

Coefficients:

Estimate Std. Error t value Pr(>|t|)

(Intercept) 9.31661 1.42987 6.516 0.0969 .

Basic_drink 0.45941 0.05635 8.153 0.0777 .

Basic_sanit -0.16659 0.03442 -4.840 0.1297

---

Signif. codes: 0 ‘***’ 0.001 ‘**’ 0.01 ‘*’ 0.05 ‘.’ 0.1 ‘ ’ 1

Residual standard error: 0.0006945 on 1 degrees of freedom

(14 observations deleted due to missingness)

Multiple R-squared: 1, Adjusted R-squared: 1

F-statistic: 1.06e+05 on 2 and 1 DF, p-value: 0.002172

**Burundi**

Call:

lm(formula = Basic_hyg ~ Basic_drink + Basic_sanit, data = Burundi)

Residuals:

Min 1Q Median 3Q Max

-0.061650 -0.015422 -0.005485 0.012720 0.082550

Coefficients:

Estimate Std. Error t value Pr(>|t|)

(Intercept) -1.072e+02 2.456e+01 -4.364 0.00181 **

Basic_drink 6.245e-02 8.276e-03 7.547 3.52e-05 ***

Basic_sanit 2.390e+00 5.277e-01 4.529 0.00143 **

---

Signif. codes: 0 ‘***’ 0.001 ‘**’ 0.01 ‘*’ 0.05 ‘.’ 0.1 ‘ ’ 1

Residual standard error: 0.04236 on 9 degrees of freedom

(6 observations deleted due to missingness)

Multiple R-squared: 0.8664, Adjusted R-squared: 0.8367

F-statistic: 29.17 on 2 and 9 DF, p-value: 0.0001166

**Cameroon**

Call:

lm(formula = Basic_hyg ~ Basic_drink + Basic_sanit, data = Cameroon)

Residuals:

15 16 17 18

-3.881e-03 6.802e-03 -2.963e-03 4.173e-05

Coefficients:

Estimate Std. Error t value Pr(>|t|)

(Intercept) 45.0431 4.8982 9.196 0.0690 .

Basic_drink -2.0447 0.1594 -12.824 0.0495 *

Basic_sanit 2.2471 0.1361 16.509 0.0385 *

---

Signif. codes: 0 ‘***’ 0.001 ‘**’ 0.01 ‘*’ 0.05 ‘.’ 0.1 ‘ ’ 1

Residual standard error: 0.008373 on 1 degrees of freedom

(14 observations deleted due to missingness)

Multiple R-squared: 0.9971, Adjusted R-squared: 0.9914

F-statistic: 174.2 on 2 and 1 DF, p-value: 0.05349

Call:

lm(formula = Basic_hyg ~ Basic_drink + Basic_sanit, data = CAF)

Residuals:

Min 1Q Median 3Q Max

-0.0073082 -0.0006677 -0.0004172 0.0020729 0.0045073

**Central African Republic**

Coefficients:

Estimate Std. Error t value Pr(>|t|)

(Intercept) 7.66032 0.77080 9.938 6.00e-05 ***

Basic_drink 0.11046 0.01029 10.733 3.86e-05 ***

Basic_sanit 0.15322 0.01195 12.817 1.39e-05 ***

---

Signif. codes: 0 ‘***’ 0.001 ‘**’ 0.01 ‘*’ 0.05 ‘.’ 0.1 ‘ ’ 1

Residual standard error: 0.004353 on 6 degrees of freedom

(9 observations deleted due to missingness)

Multiple R-squared: 0.995, Adjusted R-squared: 0.9933

F-statistic: 593.4 on 2 and 6 DF, p-value: 1.273e-07

**Chad**

Call:

lm(formula = Basic_hyg ~ Basic_drink + Basic_sanit, data = Chad)

Residuals:

12 13 14 15 16 17 18

-0.001036 0.006164 -0.004833 -0.005030 0.005573 -0.002022 0.001184

Coefficients:

Estimate Std. Error t value Pr(>|t|)

(Intercept) 25.9433 6.3791 4.067 0.0153 *

Basic_drink -0.5732 0.1874 -3.058 0.0377 *

Basic_sanit 0.2466 0.1054 2.340 0.0794 .

---

Signif. codes: 0 ‘***’ 0.001 ‘**’ 0.01 ‘*’ 0.05 ‘.’ 0.1 ‘ ’ 1

Residual standard error: 0.005574 on 4 degrees of freedom

(11 observations deleted due to missingness)

Multiple R-squared: 0.9905, Adjusted R-squared: 0.9858

F-statistic: 208.6 on 2 and 4 DF, p-value: 9.019e-05

**Comoros**

Call:

lm(formula = Basic_hyg ~ Basic_drink + Basic_sanit, data = Comoros)

Residuals:

15 16 17 18

-7.636e-05 -1.748e-04 5.808e-04 -3.296e-04

Coefficients:

Estimate Std. Error t value Pr(>|t|)

(Intercept) 9.31661 1.42987 6.516 0.0969 .

Basic_drink 0.45941 0.05635 8.153 0.0777 .

Basic_sanit -0.16659 0.03442 -4.840 0.1297

---

Signif. codes: 0 ‘***’ 0.001 ‘**’ 0.01 ‘*’ 0.05 ‘.’ 0.1 ‘ ’ 1

Residual standard error: 0.0006945 on 1 degrees of freedom

(14 observations deleted due to missingness)

Multiple R-squared: 1, Adjusted R-squared: 1

F-statistic: 1.06e+05 on 2 and 1 DF, p-value: 0.002172

**Congo**

Call:

lm(formula = Basic_hyg ~ Basic_drink + Basic_sanit, data = Congo)

Residuals:

15 16 17 18

-4.987e-05 1.309e-04 -1.124e-04 3.144e-05

Coefficients:

Estimate Std. Error t value Pr(>|t|)

(Intercept) 41.164322 0.321154 128.176 0.00497 **

Basic_drink 0.081129 0.008246 9.838 0.06449 .

Basic_sanit 0.042408 0.014018 3.025 0.20324

---

Signif. codes: 0 ‘***’ 0.001 ‘**’ 0.01 ‘*’ 0.05 ‘.’ 0.1 ‘ ’ 1

Residual standard error: 0.0001823 on 1 degrees of freedom

(14 observations deleted due to missingness)

Multiple R-squared: 1, Adjusted R-squared: 1

F-statistic: 9.098e+05 on 2 and 1 DF, p-value: 0.0007413

**Congo DRC**

Call:

lm(formula = Basic_hyg ~ Basic_drink + Basic_sanit, data = Congo_DRC)

Residuals:

Min 1Q Median 3Q Max

-0.0082869 -0.0037712 -0.0008292 0.0019072 0.0155656

Coefficients:

Estimate Std. Error t value Pr(>|t|)

(Intercept) 0.887056 1.002945 0.884 0.395

Basic_drink 0.049248 0.001287 38.258 4.71e-13 ***

Basic_sanit 0.070278 0.046922 1.498 0.162

---

Signif. codes: 0 ‘***’ 0.001 ‘**’ 0.01 ‘*’ 0.05 ‘.’ 0.1 ‘ ’ 1

Residual standard error: 0.006698 on 11 degrees of freedom

(4 observations deleted due to missingness)

Multiple R-squared: 0.997, Adjusted R-squared: 0.9965

F-statistic: 1839 on 2 and 11 DF, p-value: 1.288e-14

**Côte d'Ivoire**

Call:

lm(formula = Basic_hyg ~ Basic_drink + Basic_sanit, data = Cote_Ivoire)

Residuals:

Min 1Q Median 3Q Max

-0.34968 -0.05164 -0.00293 0.07149 0.26932

Coefficients:

Estimate Std. Error t value Pr(>|t|)

(Intercept) -535.0235 152.8184 -3.501 0.00671 **

Basic_drink 8.1496 2.2521 3.619 0.00558 **

Basic_sanit -1.2335 0.3542 -3.482 0.00692 **

---

Signif. codes: 0 ‘***’ 0.001 ‘**’ 0.01 ‘*’ 0.05 ‘.’ 0.1 ‘ ’ 1

Residual standard error: 0.1719 on 9 degrees of freedom

(6 observations deleted due to missingness)

Multiple R-squared: 0.6757, Adjusted R-squared: 0.6037

F-statistic: 9.378 on 2 and 9 DF, p-value: 0.006295

**Equatorial Guinea**

Call:

lm(formula = Basic_hyg ~ Basic_drink + Basic_sanit, data = Equatorial_Guinea)

Residuals:

Min 1Q Median 3Q Max

-0.004410 -0.002084 -0.001683 0.002624 0.006785

Coefficients:

Estimate Std. Error t value Pr(>|t|)

(Intercept) 30.20815 0.35874 84.21 1.89e-10 ***

Basic_drink 0.60051 0.01127 53.28 2.93e-09 ***

Basic_sanit -0.66208 0.01628 -40.67 1.48e-08 ***

---

Signif. codes: 0 ‘***’ 0.001 ‘**’ 0.01 ‘*’ 0.05 ‘.’ 0.1 ‘ ’ 1

Residual standard error: 0.004009 on 6 degrees of freedom

(9 observations deleted due to missingness)

Multiple R-squared: 0.9999, Adjusted R-squared: 0.9999

F-statistic: 3.584e+04 on 2 and 6 DF, p-value: 5.861e-13

**Eswatini**

Call:

lm(formula = Basic_hyg ~ Basic_drink + Basic_sanit, data = Eswatini)

Residuals:

Min 1Q Median 3Q Max

-0.31520 -0.12424 0.03739 0.13117 0.24049

Coefficients:

Estimate Std. Error t value Pr(>|t|)

(Intercept) 166.201 74.279 2.238 0.0469 *

Basic_drink 2.945 1.492 1.973 0.0741 .

Basic_sanit -5.918 3.036 -1.950 0.0772 .

---

Signif. codes: 0 ‘***’ 0.001 ‘**’ 0.01 ‘*’ 0.05 ‘.’ 0.1 ‘ ’ 1

Residual standard error: 0.2001 on 11 degrees of freedom

(4 observations deleted due to missingness)

Multiple R-squared: 0.5058, Adjusted R-squared: 0.4159

F-statistic: 5.628 on 2 and 11 DF, p-value: 0.02073

**Ethiopia**

Call:

lm(formula = Basic_hyg ~ Basic_drink + Basic_sanit, data = Ethiopia)

Residuals:

15 16 17 18

-0.0004981 0.0020466 -0.0026205 0.0010720

Coefficients:

Estimate Std. Error t value Pr(>|t|)

(Intercept) 5.56469 0.38329 14.518 0.0438 *

Basic_drink -0.04101 0.05497 -0.746 0.5919

Basic_sanit 0.55713 0.25670 2.170 0.2749

---

Signif. codes: 0 ‘***’ 0.001 ‘**’ 0.01 ‘*’ 0.05 ‘.’ 0.1 ‘ ’ 1

Residual standard error: 0.003529 on 1 degrees of freedom

(14 observations deleted due to missingness)

Multiple R-squared: 0.9996, Adjusted R-squared: 0.9989

F-statistic: 1384 on 2 and 1 DF, p-value: 0.01901

**Gabon**

Call:

lm(formula = Basic_hyg ~ Basic_drink + Basic_sanit, data = Ethiopia)

Residuals:

15 16 17 18

-0.0004981 0.0020466 -0.0026205 0.0010720

Coefficients:

Estimate Std. Error t value Pr(>|t|)

(Intercept) 5.56469 0.38329 14.518 0.0438 *

Basic_drink -0.04101 0.05497 -0.746 0.5919

Basic_sanit 0.55713 0.25670 2.170 0.2749

---

Signif. codes: 0 ‘***’ 0.001 ‘**’ 0.01 ‘*’ 0.05 ‘.’ 0.1 ‘ ’ 1

Residual standard error: 0.003529 on 1 degrees of freedom

(14 observations deleted due to missingness)

Multiple R-squared: 0.9996, Adjusted R-squared: 0.9989

F-statistic: 1384 on 2 and 1 DF, p-value: 0.01901

**Gambia**

Call:

lm(formula = Basic_hyg ~ Basic_drink + Basic_sanit, data = Gambia)

Residuals:

Min 1Q Median 3Q Max

-0.057233 -0.014678 0.009628 0.016056 0.050347

Coefficients:

Estimate Std. Error t value Pr(>|t|)

(Intercept) -5.06771 4.80158 -1.055 0.31386

Basic_drink 0.18542 0.05670 3.270 0.00746 **

Basic_sanit -0.03986 0.01040 -3.833 0.00278 **

---

Signif. codes: 0 ‘***’ 0.001 ‘**’ 0.01 ‘*’ 0.05 ‘.’ 0.1 ‘ ’ 1

Residual standard error: 0.033 on 11 degrees of freedom

(4 observations deleted due to missingness)

Multiple R-squared: 0.9911, Adjusted R-squared: 0.9895

F-statistic: 615.8 on 2 and 11 DF, p-value: 5.117e-12

**Ghana**

Call:

lm(formula = Basic_hyg ~ Basic_drink + Basic_sanit, data = Ghana)

Residuals:

15 16 17 18

0.0025 -0.0025 -0.0025 0.0025

Coefficients:

Estimate Std. Error t value Pr(>|t|)

(Intercept) 66.1010 59.3390 1.114 0.466

Basic_drink -0.5000 1.1180 -0.447 0.732

Basic_sanit 0.8485 1.7177 0.494 0.708

Residual standard error: 0.005 on 1 degrees of freedom

(13 observations deleted due to missingness)

Multiple R-squared: 0.9982, Adjusted R-squared: 0.9947

F-statistic: 281 on 2 and 1 DF, p-value: 0.04214

**Guinea**

Call:

lm(formula = Basic_hyg ~ Basic_drink + Basic_sanit, data = Guinea)

Residuals:

15 16 17 18

-0.0004392 0.0010424 -0.0007725 0.0001693

Coefficients:

Estimate Std. Error t value Pr(>|t|)

(Intercept) 40.60685 6.57627 6.175 0.102

Basic_drink -0.36217 0.09785 -3.701 0.168

Basic_sanit -0.03253 0.02285 -1.424 0.390

Residual standard error: 0.00138 on 1 degrees of freedom

(14 observations deleted due to missingness)

Multiple R-squared: 0.9998, Adjusted R-squared: 0.9994

F-statistic: 2434 on 2 and 1 DF, p-value: 0.01433

**Guinea Bissau**

Call:

lm(formula = Basic_hyg ~ Basic_drink + Basic_sanit, data = Guinea_Bissau)

Residuals:

Min 1Q Median 3Q Max

-0.114498 -0.022969 -0.001908 0.027911 0.101754

Coefficients:

Estimate Std. Error t value Pr(>|t|)

(Intercept) -24.7403 7.3498 -3.366 0.00630 **

Basic_drink 0.8247 0.1960 4.208 0.00146 **

Basic_sanit -1.1615 0.2793 -4.158 0.00160 **

---

Signif. codes: 0 ‘***’ 0.001 ‘**’ 0.01 ‘*’ 0.05 ‘.’ 0.1 ‘ ’ 1

Residual standard error: 0.06065 on 11 degrees of freedom

(4 observations deleted due to missingness)

Multiple R-squared: 0.6597, Adjusted R-squared: 0.5979

F-statistic: 10.66 on 2 and 11 DF, p-value: 0.00266

**Kenya**

Call:

lm(formula = Basic_hyg ~ Basic_drink + Basic_sanit, data = Kenya)

Residuals:

15 16 17 18

-3.730e-17 -1.667e-03 3.333e-03 -1.667e-03

Coefficients:

Estimate Std. Error t value Pr(>|t|)

(Intercept) 74.3100 99.6086 0.746 0.592

Basic_drink -0.3333 0.7454 -0.447 0.732

Basic_sanit -1.0333 1.9171 -0.539 0.685

Residual standard error: 0.004082 on 1 degrees of freedom

(14 observations deleted due to missingness)

Multiple R-squared: 0.9983, Adjusted R-squared: 0.9948

F-statistic: 290.5 on 2 and 1 DF, p-value: 0.04145

**Liberia**

Call:

lm(formula = Basic_hyg ~ Basic_drink + Basic_sanit, data = Liberia)

Residuals:

Min 1Q Median 3Q Max

-0.003086 -0.002776 0.002028 0.002222 0.002484

Coefficients:

Estimate Std. Error t value Pr(>|t|)

(Intercept) 0.25656 0.85417 0.300 0.774

Basic_drink 0.02591 0.05901 0.439 0.676

Basic_sanit -0.05649 0.20331 -0.278 0.790

Residual standard error: 0.003102 on 6 degrees of freedom

(9 observations deleted due to missingness)

Multiple R-squared: 0.9629, Adjusted R-squared: 0.9505

F-statistic: 77.85 on 2 and 6 DF, p-value: 5.11e-05

**Lesotho**

Call:

lm(formula = Basic_hyg ~ Basic_drink + Basic_sanit, data = Lesotho)

Residuals:

11 12 13 14 15 16 17

-0.0040614 0.0027569 0.0001005 0.0077891 -0.0040560 -0.0054544 0.0035118

18

-0.0005865

Coefficients:

Estimate Std. Error t value Pr(>|t|)

(Intercept) 1.466123 4.815581 0.304 0.7730

Basic_drink 0.001967 0.072712 0.027 0.9795

Basic_sanit 0.012151 0.004218 2.881 0.0346 *

---

Signif. codes: 0 ‘***’ 0.001 ‘**’ 0.01 ‘*’ 0.05 ‘.’ 0.1 ‘ ’ 1

Residual standard error: 0.00536 on 5 degrees of freedom

(10 observations deleted due to missingness)

Multiple R-squared: 0.9931, Adjusted R-squared: 0.9903

F-statistic: 358.6 on 2 and 5 DF, p-value: 3.988e-06

**Madagascar**

Call:

lm(formula = Basic_hyg ~ Basic_drink + Basic_sanit, data = Madagascar)

Residuals:

ALL 3 residuals are 0: no residual degrees of freedom!

Coefficients:

Estimate Std. Error t value Pr(>|t|)

(Intercept) 21.083 NA NA NA

Basic_drink 1.159 NA NA NA

Basic_sanit -3.159 NA NA NA

Residual standard error: NaN on 0 degrees of freedom

(2 observations deleted due to missingness)

Multiple R-squared: 1, Adjusted R-squared: NaN

F-statistic: NaN on 2 and 0 DF, p-value: NA

**Mali**

Call:

lm(formula = Basic_hyg ~ Basic_drink + Basic_sanit, data = Mali)

Residuals:

15 16 17 18

0.0003969 -0.0019634 0.0027313 -0.0011648

Coefficients:

Estimate Std. Error t value Pr(>|t|)

(Intercept) 38.42304 2.96466 12.960 0.049 *

Basic_drink 0.21185 0.08263 2.564 0.237

Basic_sanit -0.07045 0.08908 -0.791 0.574

---

Signif. codes: 0 ‘***’ 0.001 ‘**’ 0.01 ‘*’ 0.05 ‘.’ 0.1 ‘ ’ 1

Residual standard error: 0.003582 on 1 degrees of freedom

(14 observations deleted due to missingness)

Multiple R-squared: 1, Adjusted R-squared: 0.9999

F-statistic: 1.189e+04 on 2 and 1 DF, p-value: 0.006485

**Mauritania**

Call:

lm(formula = Basic_hyg ~ Basic_drink + Basic_sanit, data = Mauritania)

Residuals:

15 16 17 18

0.0004856 -0.0013838 0.0013112 -0.0004130

Coefficients:

Estimate Std. Error t value Pr(>|t|)

(Intercept) 29.89609 1.43025 20.903 0.0304 *

Basic_drink 0.25498 0.04244 6.008 0.1050

Basic_sanit -0.10163 0.03244 -3.133 0.1967

---

Signif. codes: 0 ‘***’ 0.001 ‘**’ 0.01 ‘*’ 0.05 ‘.’ 0.1 ‘ ’ 1

Residual standard error: 0.00201 on 1 degrees of freedom

(14 observations deleted due to missingness)

Multiple R-squared: 1, Adjusted R-squared: 1

F-statistic: 3.189e+04 on 2 and 1 DF, p-value: 0.00396

**Mozambique**

Call:

lm(formula = Basic_hyg ~ Basic_drink + Basic_sanit, data = Mozambique)

Residuals:

Min 1Q Median 3Q Max

-0.10414 -0.02905 0.01623 0.01951 0.08078

Coefficients:

Estimate Std. Error t value Pr(>|t|)

(Intercept) 11.8395 0.3745 31.615 6.65e-08 ***

Basic_drink -1.2889 0.3744 -3.442 0.0138 *

Basic_sanit 2.4705 0.7018 3.520 0.0125 *

---

Signif. codes: 0 ‘***’ 0.001 ‘**’ 0.01 ‘*’ 0.05 ‘.’ 0.1 ‘ ’ 1

Residual standard error: 0.05939 on 6 degrees of freedom

(9 observations deleted due to missingness)

Multiple R-squared: 0.9328, Adjusted R-squared: 0.9105

F-statistic: 41.67 on 2 and 6 DF, p-value: 0.0003028

Call:

lm(formula = Basic_hyg ~ Basic_drink + Basic_sanit, data = Mozambique)

Residuals:

8 9 10 11 12 13 14

-0.0023007 0.0044010 -0.0013908 -0.0007961 0.0009186 -0.0021000 0.0012680

Coefficients:

Estimate Std. Error t value Pr(>|t|)

(Intercept) 11.31019 0.02342 483.022 1.1e-10 ***

Basic_drink -0.11199 0.03446 -3.250 0.0314 *

Basic_sanit 0.23666 0.06512 3.634 0.0221 *

---

Signif. codes: 0 ‘***’ 0.001 ‘**’ 0.01 ‘*’ 0.05 ‘.’ 0.1 ‘ ’ 1

Residual standard error: 0.002919 on 4 degrees of freedom

(9 observations deleted due to missingness)

Multiple R-squared: 0.9986, Adjusted R-squared: 0.9979

F-statistic: 1416 on 2 and 4 DF, p-value: 1.989e-06

**Namibia**

Call:

lm(formula = Basic_hyg ~ Basic_drink + Basic_sanit, data = Namibia)

Residuals:

Min 1Q Median 3Q Max

-0.024464 -0.004350 0.002983 0.011645 0.014048

Coefficients:

Estimate Std. Error t value Pr(>|t|)

(Intercept) -85.1536 10.7509 -7.921 0.000215 ***

Basic_drink 1.9351 0.2242 8.630 0.000133 ***

Basic_sanit -0.8687 0.2247 -3.866 0.008309 **

---

Signif. codes: 0 ‘***’ 0.001 ‘**’ 0.01 ‘*’ 0.05 ‘.’ 0.1 ‘ ’ 1

Residual standard error: 0.01648 on 6 degrees of freedom

(9 observations deleted due to missingness)

Multiple R-squared: 0.9998, Adjusted R-squared: 0.9997

F-statistic: 1.505e+04 on 2 and 6 DF, p-value: 7.921e-12

**Niger**

Call:

lm(formula = Basic_hyg ~ Basic_drink + Basic_sanit, data = Niger)

Residuals:

Min 1Q Median 3Q Max

-0.008444 -0.003638 0.001283 0.002559 0.007450

Coefficients:

Estimate Std. Error t value Pr(>|t|)

(Intercept) 3.07191 1.00649 3.052 0.02245 *

Basic_drink 0.20311 0.03727 5.450 0.00159 **

Basic_sanit -0.26975 0.06220 -4.337 0.00489 **

---

Signif. codes: 0 ‘***’ 0.001 ‘**’ 0.01 ‘*’ 0.05 ‘.’ 0.1 ‘ ’ 1

Residual standard error: 0.006699 on 6 degrees of freedom

(9 observations deleted due to missingness)

Multiple R-squared: 0.9967, Adjusted R-squared: 0.9956

F-statistic: 913 on 2 and 6 DF, p-value: 3.513e-08

**Nigeria**

Call:

lm(formula = Basic_hyg ~ Basic_drink + Basic_sanit, data = Nigeria)

Residuals:

15 16 17 18

-0.0011889 0.0027116 -0.0018588 0.0003362

Coefficients:

Estimate Std. Error t value Pr(>|t|)

(Intercept) 32.39329 0.10429 310.609 0.00205 **

Basic_drink 0.21779 0.08137 2.677 0.22761

Basic_sanit -0.15291 0.15001 -1.019 0.49389

---

Signif. codes: 0 ‘***’ 0.001 ‘**’ 0.01 ‘*’ 0.05 ‘.’ 0.1 ‘ ’ 1

Residual standard error: 0.003512 on 1 degrees of freedom

(14 observations deleted due to missingness)

Multiple R-squared: 0.9999, Adjusted R-squared: 0.9998

F-statistic: 6788 on 2 and 1 DF, p-value: 0.008582

**Sao Tome and Principe**

Call:

lm(formula = Basic_hyg ~ Basic_drink + Basic_sanit, data = Sao_Tome)

Residuals:

11 12 13 14 15 16 17 18

-0.091504 -0.005837 0.080791 0.177667 -0.115457 -0.049790 0.029711 -0.025581

Coefficients:

Estimate Std. Error t value Pr(>|t|)

(Intercept) 44.47684 12.58540 3.534 0.0167 *

Basic_drink 0.02476 0.25109 0.099 0.9253

Basic_sanit -0.12097 0.20146 -0.600 0.5744

---

Signif. codes: 0 ‘***’ 0.001 ‘**’ 0.01 ‘*’ 0.05 ‘.’ 0.1 ‘ ’ 1

Residual standard error: 0.113 on 5 degrees of freedom

(10 observations deleted due to missingness)

Multiple R-squared: 0.9262, Adjusted R-squared: 0.8967

F-statistic: 31.38 on 2 and 5 DF, p-value: 0.001479

**Senegal**

Call:

lm(formula = Basic_hyg ~ Basic_drink + Basic_sanit, data = Senegal)

Residuals:

15 16 17 18

3.721e-28 -9.419e-16 1.884e-15 -9.419e-16

Coefficients:

Estimate Std. Error t value Pr(>|t|)

(Intercept) 1.715e+01 1.149e-12 1.492e+13 4.27e-14 ***

Basic_drink 4.057e-01 1.202e-13 3.377e+12 1.89e-13 ***

Basic_sanit -5.000e-01 2.106e-13 -2.374e+12 2.68e-13 ***

---

Signif. codes: 0 ‘***’ 0.001 ‘**’ 0.01 ‘*’ 0.05 ‘.’ 0.1 ‘ ’ 1

Residual standard error: 2.307e-15 on 1 degrees of freedom

(13 observations deleted due to missingness)

Multiple R-squared: 1, Adjusted R-squared: 1

F-statistic: 1.015e+28 on 2 and 1 DF, p-value: 7.018e-15

**Sierra Leone**

Call:

lm(formula = Basic_hyg ~ Basic_drink + Basic_sanit, data = Sierra_Leone)

Residuals:

7 8 9 10 11 12 13

0.0035148 -0.0037585 -0.0010319 -0.0019013 -0.0002825 0.0046982 0.0021671

14

-0.0034061

Coefficients:

Estimate Std. Error t value Pr(>|t|)

(Intercept) 11.91171 0.03895 305.847 7.09e-12 ***

Basic_drink -0.05539 0.02723 -2.034 0.0976 .

Basic_sanit 0.30421 0.10459 2.908 0.0335 *

---

Signif. codes: 0 ‘***’ 0.001 ‘**’ 0.01 ‘*’ 0.05 ‘.’ 0.1 ‘ ’ 1

Residual standard error: 0.003731 on 5 degrees of freedom

(6 observations deleted due to missingness)

Multiple R-squared: 0.9983, Adjusted R-squared: 0.9976

F-statistic: 1448 on 2 and 5 DF, p-value: 1.232e-07

**Somalia**

Call:

lm(formula = Basic_hyg ~ Basic_drink + Basic_sanit, data = Somalia)

Residuals:

12 13 14 15 16 17 18

0.024972 -0.018582 -0.057071 0.040484 0.024083 0.001711 -0.015597

Coefficients:

Estimate Std. Error t value Pr(>|t|)

(Intercept) 8.17615 0.32997 24.779 1.57e-05 ***

Basic_drink 0.10348 0.14595 0.709 0.517

Basic_sanit -0.09799 0.19364 -0.506 0.639

---

Signif. codes: 0 ‘***’ 0.001 ‘**’ 0.01 ‘*’ 0.05 ‘.’ 0.1 ‘ ’ 1

Residual standard error: 0.0409 on 4 degrees of freedom

(11 observations deleted due to missingness)

Multiple R-squared: 0.9134, Adjusted R-squared: 0.8701

F-statistic: 21.1 on 2 and 4 DF, p-value: 0.007495

**South Africa**

Call:

lm(formula = Basic_hyg ~ Basic_drink + Basic_sanit, data = South_Africa)

Residuals:

15 16 17 18

-0.0011500 0.0017931 -0.0001237 -0.0005194

Coefficients:

Estimate Std. Error t value Pr(>|t|)

(Intercept) 83.6371 30.3528 2.755 0.222

Basic_drink -0.8479 0.5160 -1.643 0.348

Basic_sanit 0.5140 0.2306 2.229 0.268

Residual standard error: 0.002196 on 1 degrees of freedom

(14 observations deleted due to missingness)

Multiple R-squared: 0.9999, Adjusted R-squared: 0.9998

F-statistic: 8231 on 2 and 1 DF, p-value: 0.007794

**Sudan**

Call:

lm(formula = Basic_hyg ~ Basic_drink + Basic_sanit, data = Sudan)

Residuals:

11 12 13 14 15 16 17 18

0.002328 -0.003012 -0.001535 -0.001848 0.006248 0.001161 -0.017991 0.014649

Coefficients:

Estimate Std. Error t value Pr(>|t|)

(Intercept) 25.99509 1.19137 21.82 3.75e-06 ***

Basic_drink -0.13921 0.05081 -2.74 0.0408 *

Basic_sanit 0.15912 0.05133 3.10 0.0269 *

---

Signif. codes: 0 ‘***’ 0.001 ‘**’ 0.01 ‘*’ 0.05 ‘.’ 0.1 ‘ ’ 1

Residual standard error: 0.01094 on 5 degrees of freedom

(10 observations deleted due to missingness)

Multiple R-squared: 0.9761, Adjusted R-squared: 0.9666

F-statistic: 102.2 on 2 and 5 DF, p-value: 8.805e-05

**Tanzania**

Call:

lm(formula = Basic_hyg ~ Basic_drink + Basic_sanit, data = Tanzania)

Residuals:

13 14 15 16 17 18

-2.041e-04 3.539e-04 6.484e-06 -1.501e-04 -1.157e-04 1.095e-04

Coefficients:

Estimate Std. Error t value Pr(>|t|)

(Intercept) 45.18264 0.18338 246.386 1.47e-07 ***

Basic_drink -0.01909 0.00912 -2.093 0.1274

Basic_sanit 0.12873 0.01116 11.532 0.0014 **

---

Signif. codes: 0 ‘***’ 0.001 ‘**’ 0.01 ‘*’ 0.05 ‘.’ 0.1 ‘ ’ 1

Residual standard error: 0.0002676 on 3 degrees of freedom

(12 observations deleted due to missingness)

Multiple R-squared: 1, Adjusted R-squared: 1

F-statistic: 3.128e+06 on 2 and 3 DF, p-value: 3.322e-10

**Togo**

Call:

lm(formula = Basic_hyg ~ Basic_drink + Basic_sanit, data = Togo)

Residuals:

Min 1Q Median 3Q Max

-0.0070418 -0.0030807 -0.0004505 0.0017141 0.0069658

Coefficients:

Estimate Std. Error t value Pr(>|t|)

(Intercept) 7.48188 0.21387 34.983 8.64e-12 ***

Basic_drink -0.01855 0.01118 -1.659 0.128

Basic_sanit 0.26041 0.03204 8.127 1.03e-05 ***

---

Signif. codes: 0 ‘***’ 0.001 ‘**’ 0.01 ‘*’ 0.05 ‘.’ 0.1 ‘ ’ 1

Residual standard error: 0.004543 on 10 degrees of freedom

(5 observations deleted due to missingness)

Multiple R-squared: 0.9998, Adjusted R-squared: 0.9998

F-statistic: 2.914e+04 on 2 and 10 DF, p-value: < 2.2e-16

**Tunisia**

Call:

lm(formula = Basic_hyg ~ Basic_drink + Basic_sanit, data = Tunisia)

Residuals:

9 10 11 12 13 14

-9.948e-07 1.114e-04 -1.617e-04 1.052e-04 -1.678e-04 1.139e-04

Coefficients:

Estimate Std. Error t value Pr(>|t|)

(Intercept) 8.125e+01 2.252e-02 3606.89 4.70e-11 ***

Basic_drink 1.482e-03 9.628e-04 1.54 0.221

Basic_sanit 5.399e-02 7.786e-04 69.34 6.61e-06 ***

---

Signif. codes: 0 ‘***’ 0.001 ‘**’ 0.01 ‘*’ 0.05 ‘.’ 0.1 ‘ ’ 1

Residual standard error: 0.0001739 on 3 degrees of freedom

(8 observations deleted due to missingness)

Multiple R-squared: 1, Adjusted R-squared: 1

F-statistic: 7.232e+05 on 2 and 3 DF, p-value: 2.987e-09

**Zambia**

Call:

lm(formula = Basic_hyg ~ Basic_drink + Basic_sanit, data = Zambia)

Residuals:

Min 1Q Median 3Q Max

-0.09466 -0.01475 0.01654 0.02415 0.05758

Coefficients:

Estimate Std. Error t value Pr(>|t|)

(Intercept) 8.4241 8.0350 1.048 0.3348

Basic_drink 0.3617 0.1560 2.319 0.0595 .

Basic_sanit -0.6143 0.6586 -0.933 0.3870

---

Signif. codes: 0 ‘***’ 0.001 ‘**’ 0.01 ‘*’ 0.05 ‘.’ 0.1 ‘ ’ 1

Residual standard error: 0.05653 on 6 degrees of freedom

(9 observations deleted due to missingness)

Multiple R-squared: 0.9805, Adjusted R-squared: 0.974

F-statistic: 150.7 on 2 and 6 DF, p-value: 7.441e-06

**Zimbabwe**

Call:

lm(formula = Basic_hyg ~ Basic_drink + Basic_sanit, data = Zimbabwe)

Residuals:

15 16 17 18

-9.090e-16 1.818e-15 -9.090e-16 7.430e-29

Coefficients:

Estimate Std. Error t value Pr(>|t|)

(Intercept) 6.862e+00 1.210e-11 5.672e+11 1.12e-12 ***

Basic_drink 7.500e-01 3.127e-13 2.398e+12 2.65e-13 ***

Basic_sanit -5.000e-01 2.190e-13 -2.283e+12 2.79e-13 ***

---

Signif. codes: 0 ‘***’ 0.001 ‘**’ 0.01 ‘*’ 0.05 ‘.’ 0.1 ‘ ’ 1

Residual standard error: 2.227e-15 on 1 degrees of freedom

(14 observations deleted due to missingness)

Multiple R-squared: 1, Adjusted R-squared: 1

F-statistic: 1.488e+26 on 2 and 1 DF, p-value: 5.797e-14

**MAIN R FUNCTIONS**

#Perform a PCA on the dataframe called "mydata"

mydata<- prcomp(na.omit(mydata), center = TRUE, scale. = TRUE)

summary(mydata)

#Take the four first components of the PCA

comp<- data.frame(mydata$x[,1:4])

#Find clusters (HCDC)

library(FactoMineR)

library(factoextra)

res.hcpc <- HCPC(comp, graph = FALSE)

mydata<-cbind(cluster=res.hcpc$data.clust[4],mydata)

#Decision tree considering the values of the dummy variable for both population groups ("LRI_ov5" and "LRI_un5") as explanatory variables and the country group ("A", "B", "C") as the dependent one.

library(rpart)

library(rpart.plot)

library(caret)

fit <- rpart(Group ~ LRI_ov5+LRI_un5, method="class", data=df, weights=df$Weight)

rpart.plot(fit, box.palette=list('#1E90FF','#32CD32','#FA8072'))

pred <- predict(object = fit, newdata = df, type = "class")

df2<-cbind(df,pred)

confusionMatrix(table(df2$pred, df2$Group))

#Structure learning Bayesian network

library(bnlearn)

library(visNetwork)

library(arules)

df<-na.omit(mydata)

prueba <- subset(df, select = c("Pop_dens", "Pop_urb", ""Basic_hyg", "GDP_cap", "M_remitt_capita", "ODA_capita", "Pop_age", "Pop_HIV", "Mort_rate_LRI"))

set.seed(123)

hill=hc(prueba)

plot(hill)

arc.strength(hill,prueba) #Arc strength assessment

set.seed(123)

hill=hc(prueba, blacklist = blacklist, whitelist=whitelist) #Structure learning considering a blacklist and a whitelist of arcs (according to arc strength and previous knowledge)

bn.cv(prueba,hill) #Score

mm_hill=mmhc(prueba, blacklist = blacklist, whitelist=whitelist) #Structure learning (Max-Min Hill Climbing)

bn.cv(prueba,mm_hill) #Score

iamb_alg=iamb(prueba, blacklist = blacklist, whitelist=whitelist) #Structure learning (Incremental Association Markov Blanket)

bn.cv(prueba,iamb_alg) #Score

tabus=tabu(prueba, blacklist = blacklist, whitelist=whitelist) #Structure learning (Tabu Search)

bn.cv(prueba,tabus) #Score

gs_alg=gs(prueba, blacklist = blacklist, whitelist=whitelist) #Structure learning (Grow Shrink)

bn.cv(prueba,gs_alg) #Score

#Making inferences based on the selected structure and discretized data ("class.df")

set.seed(123)

structure <- empty.graph(c("GDP_cap", "M_remitt_capita", "ODA_capita", "Pop_urb", "Pop_dens", "ODA_WASH_cap", "Basic_hyg", "Mort_rate_LRI", "Pop_HIV", "Pop_age"))

modelstring(structure) <- "[Pop_dens][GDP_cap][M_remitt_capita][ODA_capita][ODA_WASH_cap|ODA_capita][Pop_HIV][Pop_urb|Pop_dens:GDP_cap][Basic_hyg|Pop_urb:M_remitt_capita:ODA_WASH_cap][Pop_age|Pop_dens:Basic_hyg:GDP_cap:M_remitt_capita][Mort_rate_LRI|Basic_hyg:Pop_age:Pop_HIV]"

bn.mod <- bn.fit(structure, data = class.df)

#Probability of high mortality due to LRI if population access to hygiene services is low:

cat("P(High LRI mortality rate | Low access to basic hygiene) =", cpquery(bn.mod, (Mort_rate_LRI=="High"), (Basic_hyg == "Low")), "\n")

#Belief propagation

library(dplyr)

cluster_C<-filter(prueba, Country=="Angola" | Country=="Benin" | Country=="Burkina Faso" | Country=="Burundi" | Country=="Cameroon" | Country=="Central African Republic" | Country=="Chad" | Country=="Comoros" | Country=="Congo" | Country=="Congo DRC" | Country=="Côte d'Ivoire" | Country=="Ethiopia" | Country=="Gambia" | Country=="Guinea" | Country=="Guinea-Bissau" | Country=="Kenya" | Country=="Liberia" | Country=="Madagascar" | Country=="Malawi" | Country=="Mali" | Country=="Mauritania" | Country=="Mozambique" | Country=="Niger" | Country=="Nigeria" | Country=="Rwanda" | Country=="Senegal" | Country=="Sierra Leone" | Country=="Somalia" | Country=="Sudan" | Country=="Tanzania" | Country=="Togo" | Country=="Uganda" | Country=="Zambia" | Country=="Zimbabwe")

library(BayesNetBP)

library("Rgraphviz")

library("igraph")

dag.graphNEL <- bn_to_graphNEL(structure)

node.names <- nodes(dag.graphNEL)

node.class <- rep(FALSE, length(node.names))

names(node.class) <- node.names

tree.init.p <- Initializer(dag = dag.graphNEL, data = cluster_C, node.class = node.class, propagate = TRUE)

tree.post <- AbsorbEvidence(tree.init.p, c("M_remitt_capita"), list(3)) #Scenario 1

set.seed(123)

marg <- Marginals(tree.post, c("Basic_hyg", "Mort_rate_LRI"))

SummaryMarginals(marg)

PlotCGBN(tree.init.p, tree.post)
